# Hippo Signaling Pathway Involvement in Osteopotential Regulation of Murine Bone Marrow Cells Under Simulated Microgravity

**DOI:** 10.3390/cells13221921

**Published:** 2024-11-19

**Authors:** Ekaterina Tyrina, Danila Yakubets, Elena Markina, Ludmila Buravkova

**Affiliations:** Cell Physiology Laboratory, Institute of Biomedical Problems, Russian Academy of Sciences, 123007 Moscow, Russia; lizard_96@mail.ru (D.Y.); buravkova@imbp.ru (L.B.)

**Keywords:** microgravity simulation, heterotypic bone marrow cell culture, Hippo signaling pathway, osteodifferentiation, yes-associated peptide (Yap)

## Abstract

The development of osteopenia is one of the most noticeable manifestations of the adverse effects of space factors on crew members. The Hippo signaling pathway has been shown to play a central role in regulating the functional activity of cells through their response to mechanical stimuli. In the present study, the components of the Hippo pathway and the protective properties of osteodifferentiation inducers were investigated under simulated microgravity (smg) using a heterotypic bone marrow cell culture model, which allows for the maintenance of the close interaction between the stromal and hematopoietic compartments, present in vivo and of great importance for both the fate of osteoprogenitors and hematopoiesis. After 14 days of smg, the osteopotential and osteodifferentiation of bone marrow stromal progenitor cells, the expression of Hippo cascade genes and the immunocytochemical status of the adherent fraction of bone marrow cells, as well as the paracrine profile in the conditioned medium and the localization of Yap1 and Runx2 in mechanosensitive cells of the bone marrow were obtained. Simulated microgravity negatively affects stromal and hematopoietic cells when interacting in a heterotypic murine bone marrow cell culture. This is evidenced by the decrease in cell proliferation and osteopotential. Changes in the production of pleiotropic cytokines IL-6, GROβ and MCP-1 were revealed. Fourteen days of simulated microgravity induced a decrease in the nuclear translocation of Yap1 and the transcription factor Runx2 in the stromal cells of the intact group. Exposure to osteogenic induction conditions partially compensated for the negative effect of simulated microgravity. The data obtained will be crucial for understanding the effects of spaceflight on osteoprogenitor cell growth and differentiation via Hippo–Yap signaling.

## 1. Introduction

Spaceflight factors, including microgravity, are known to have negative impacts on various human and animal physiological systems. This has been shown in numerous studies conducted in recent years [1,2,3,4,5]. One serious consequence of microgravity is osteopenia characterized by reduced bone density [6], leading to decreased bone mass and a reduction in the number of bone cells [7,8]. It has been shown that the underlying cause of osteopenia in microgravity is a change in bone homeostasis. Specifically, there is an activation of osteoclastic resorption and a slowing down of bone formation [9]. One of the reasons for this is the inhibition of the functional activity of the pool of osteogenic bone marrow mesenchymal stem cells (MSCs).

In real spaceflights and simulated microgravity, there has been a decrease in the number of bone marrow stromal progenitor cells, which are precursors of osteoblasts, and a change in their differentiation potential, i.e., an inhibition of osteodifferentiation [9,10,11,12,13]. Due to the close interaction between mesenchymal stem cells (MSCs) and hematopoietic progenitors underlying hematopoiesis, any disturbances in the stromal environment could also lead to lesions in the hematopoietic compartment.

Both extracellular and cellular mechanosensitive compartments are known to be targets for gravitational deprivation signals. Components of the extracellular matrix (ECM), cytoskeleton (CS) and nucleoskeleton (NS) can be considered gravity sensors, while transmembrane complexes (TCs) and linker of nucleoskeleton and cytoskeleton complexes (LINCs) ensure the conversion and transmission of altered gravity signals to the nucleus [14,15,16,17]. In this context, mechanosensitive signaling pathways such as Hippo are of particular interest. Many Hippo signaling components localize to cell–cell contacts (e.g., PAR6, β-catenin), and some of them have been shown to interact with the cytoskeleton, e.g., α-catenin [18,19]. Hippo signaling has affected the activity of yes-associated peptide (Yap), a transcriptional coactivator and critical effector component of the Hippo pathway [20]. Yap and the transcriptional coactivator with PDZ-binding motif (Taz) have been shown to be involved in osteogenesis [21,22,23,24,25]. Yap can act as a mechanosensor by transducing signals that control various cellular functions, including the osteocommitment of MSCs through the activation of Runx2 [26,27].

In real spaceflight and in ground-based microgravity models, levels of pro-inflammatory cytokines increase in peripheral blood and tissues [28,29]. It is well known that pro-inflammatory cytokines (IL-1, IL-6, TNF-α) inhibit the osteoblastic differentiation of MSCs, reduce the functional activity of osteoblasts and activate osteoclasts [30,31,32,33]. Elevated levels of these cytokines often accompany osteoporosis, including that associated with ageing [33]. Pro-inflammatory cytokines have been found to activate the Hippo signaling pathway, which plays a central role in regulating the functional activity of cells through their response to mechanical stimuli [34]. This leads to the inhibition of Yap, which is associated with a slowdown in the osteodifferentiation of bone marrow-derived MSCs [34]. Thus, the involvement of the Hippo cascade against the background of pro-inflammatory activation in the conditions of mechanical deprivation may lead to the alteration of intracellular homeostasis with an inhibition of MSC osteopotential.

Therefore, in this work, we analyzed the components of the Hippo pathway in mouse bone marrow microexplants under simulated microgravity in vitro. This model was chosen to preserve the close interaction between the stromal and hematopoietic compartments of the bone marrow, which is important for both the fate of osteoprogenitors and hematopoiesis.

## 2. Materials and Methods

Male BalbC mice (6 and 8 weeks old) were provided by the vivarium of the State scientific center of the Russian Federation—Institute of Biomedical Problems of the Russian Academy of Sciences (SSC RF—IBMP RAS). We followed the protocols approved by the Commission on Biomedical Ethics of the Institute of Biomedical Problems (Protocol No. 652 of 30.11.23).

### 2.1. Murine Bone Marrow Isolation

After euthanasia, bone marrow (BM) was isolated from the mice femur and tibia bones according to the generally accepted procedure with our modification [35,36]. In brief, the bones were dissected from the hindlimbs and cleaned of muscle and connective tissue. Then, the epiphyses were removed and the entire bone marrow was carefully extracted from the medullary canal using a 20 mL syringe (23 G needle) containing 20 mL of complete culture medium (α-MEM medium (Gibco, Life Technologies, Carlsbad, CA, USA) supplemented with 20% fetal bovine serum (FBS) (HyClone, Logan, UT, USA), 100 μg/mL streptomycin, 100 U/mL penicillin (PanEco, Moscow, Russia) and 2 mM glutamine (PanEco, Moscow, Russia). The procedure was repeated 3 times for complete removal of the bone marrow. The isolated bone marrow cells were collected in a 50 mL tube.

**Figure 1:**
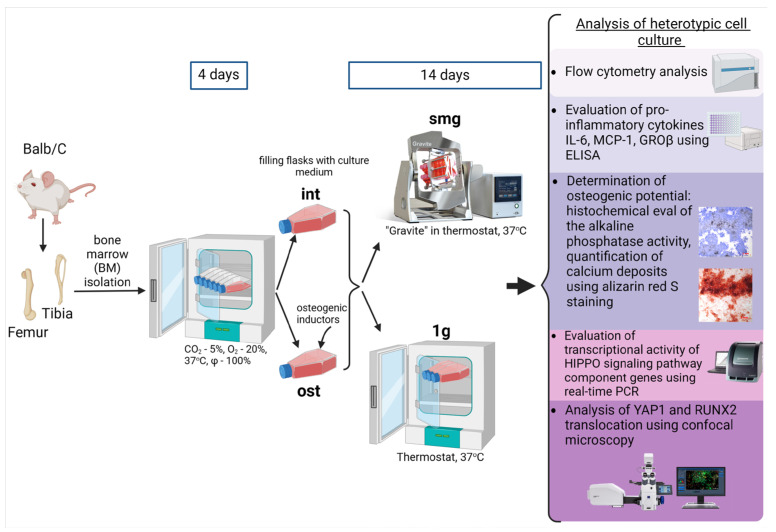
Experimental design. Bone marrow cells isolated from Balb/C femur and tibia were cultured in 25 cm^2^ flasks for 4 days according to the routine protocol. On day 5, the medium containing non-adherent cells was removed and the flasks were completely filled with a fresh culture medium—intact medium (int) and osteogenic culture medium (ost). Heterotypic bone marrow cell cultures were divided into 2 groups: 1 g—standard culture conditions; and smg—3D clinorotation for 14 days. At the end of the experiment, the cells were initiated for in vitro analysis.

After isolation and counting, cells were pooled and transferred to culture flasks (CELLSTAR, Greiner Bio One, Frickenhausen, Germany). Cells were cultured in a complete culture medium for 4 days at 37 °C, 100% humidity, 5% CO_2_ and 20% O_2_. On day 5, the medium containing unattached cells was removed. Half of the flasks were completely filled with an intact medium (int) and half with an osteogenic medium (ost) containing 10 nM dexamethasone, 10 mM β-glycerophosphate and 200 μM ascorbic acid (Sigma-Aldrich, St. Louis, MO, USA). Flasks were completely filled with the culture medium without air bubbles to prevent the sloshing of the medium and shear stress. Then, the culture medium was pre-saturated with CO_2_ (12 h), and then the flasks were sealed tight. After that, the flasks were placed in a thermostat (37 °C) in a static position (static control—1 g) or were fixed on the random positioning machine «Gravite» platform (smg) for 14 days (Figure 1).

### 2.2. The Microgravity Simulation

The microgravity simulation was carried out using a device for the random positioning of the object relative to the gravity vector ‘Gravite’ (Space Biolaboratories Co., Ltd., Hiroshima, Japan) for 14 days. This method simulates 10^−2^–10^−3^g and has been widely used to work with cell culture in vitro [37,38].

### 2.3. Enzyme-Linked Immunosorbent Assay (ELISA)

After 14 days of simulated microgravity, we collected the conditioned medium to analyze paracrine activity. The medium was centrifuged at 2500× *g* to remove cellular debris and then stored at −80 °C until measurements were taken. Both mesenchymal stromal cells and hematopoietic cells produced a range of paracrine factors. Conditioned medium samples were thawed at room temperature immediately before measurement. To assess the production of cytokines IL-6, GROβ and MCP-1, enzyme immunoassay was used using Elabscience kits (Elabscience Biotechnology Co., Ltd., Wuhan, Hubei, China) according to the manufacturer’s instructions. The optical density of samples and standards was determined at a wavelength of 450 nm on a flatbed spectrophotometer (Bio-Rad Laboratories, Hercules, CA, USA). The concentration of analytes was measured according to the created calibration curve (Figure 1).

### 2.4. Quantitative Real-Time PCR

The expression of Hippo signaling pathway genes (*Ajuba*, *Amot*, *Casp3*, *Dchs1*, *Fat4*, *Limd1*, *Lpp*, *Nf2*, *Wtip*, *Lats2*, *Mob1a*, *Mob1b*, *Sav1*, *Stk3*, *Meis1*, *Ptprz1*, *Lats1*, *Patj*, *Ywhaq*), the transcription factors Taz and Yap1 (*Wwtr1*, *Yap1*) and their mediator Tead2 (*Tead2*), Runx2 (*Runx2*), as well as the target genes of the transcription factor Yap/Taz (*Ccn*, *Itgb2*) and Runx2 (*Alpl*), was assessed by quantitative real-time PCR with reverse transcription. Primers for further quantitative real-time PCR were selected and synthesized for all these genes. The synthesized primers were ordered from Evrogen (Evrogen, Moscow, Russia) (Table 1) and tested against RNA isolated from control bone marrow microexplants. The specificity of the primers was verified by analyzing the melting curves of amplification products ranging from 55 °C to 95 °C with a 1 °C step on the MX3000P device (Stratagene, La Jolla, CA, USA).

Total RNA was isolated using ExtractRNA (Evrogen, Moscow, Russia). The concentration and purity of the obtained RNA were evaluated using a NanoDrop 2000c (Thermo Fisher Scientific Inc., Waltham, MA, USA) spectrophotometer by the absorbance level at the 260/280 nm wavelength. Genomic DNA was removed using DNAase (Qiagen Sciences LLC, Germantown, MD, USA) according to the manufacturer’s protocol. RNA was then further purified using spin columns with the CleanRNA standard kit (Evrogen, Moscow, Russia) according to the manufacturer’s protocol. The concentration and purity of the RNA were determined, and the concentrations were equalized. Subsequently, cDNA was synthesized using MMLV reverse transcriptase on the RNA matrix and the MMLV RT kit (Evrogen, Moscow, Russia) following the manufacturer’s instructions. PCR was performed with the 5X qPCRmix-HS SYBR kit (Evrogen, Moscow, Russia) according to the protocol provided by the manufacturer. Primers for *Actb* (Qiagen Sciences LLC, Germantown, MD, USA), *Gapdh* (Qiagen Sciences LLC, Germantown, MD, USA) and *Hsp90a* (Evrogen, Moscow, Russia) were used as housekeeping genes, and all genes showed stable expression levels under control and experimental conditions. The expression level was assessed using the 2^−∆∆Ct^ method [39].

### 2.5. Microscopy

Cells were analyzed using a phase-contrast microscope (Nikon Eclipse TiU, Tokyo, Japan) equipped with a camera for image capture and digitization.

To analyze the translocation of Yap1 and Runx2, bone marrow microexplants were cultured on glass slide-flasks (Nunclon Delta, Thermo Scientific Chemicals, Waltham, MA, USA). Slide associations were washed three times with phosphate-salt buffer (PBS), fixed for 10 min with 4% paraformaldehyde at 37 °C, washed three times with PBS, incubated in 0.2% Triton X-100 for 15 min, washed three times with PBS and incubated in 3% BSA for 1 h. After being washed with PBS, the cells were incubated with rabbit polyclonal antibodies (dilution of 1:100) against Yap1 (Affinity Biosciences, Cincinnati, OH, USA) or Runx2 (Elabscience Biotechnology Co., Ltd., Wuhan, Hubei, China) for 1 h at room temperature. After being washed with PBS, cells were incubated with secondary goat anti-rabbit IgG(H+L) conjugated to Elab Fluor 488 (Elabscience Biotechnology Co., Ltd., Wuhan, Hubei, China) for 1 h at room temperature. After being washed, the cells were incubated with PE-conjugated mouse anti-CD45 antibody (BD Biosciences, Franklin Lakes, NJ, USA) for 1 h. After being washed with PBS, cells were embedded in DAPI-Fluoroshield™ (Thermo Scientific Chemicals, Waltham, MA, USA) and analyzed using a Zeiss LSM 900 laser scanning microscope (Zeiss, Oberkochen, Germany). The images were exported in TIFF format per channel (blue—DAPI; green—ElabFluor488; red—PE).

More than 700 images were acquired. Further processing was conducted in CellProfiler (v.4.2.6) [40]. Implemented as a plugin, the universal cell segmentation algorithm Cellpose is used for cellular segmentation [41]. This neural network algorithm can be trained on user data, allowing the analysis of almost any cell culture type. Cellpose was trained to identify and segment large CD45− cells. The blue channel showed large nuclei. Only cells with clearly defined borders and nuclei were included in the analysis. CD45+ cells and nuclei were also segmented within regions of CD45− cells using standard CellProfiler algorithms. Large CD45− cells were masked, and the remaining field was used to segment the nuclei and borders of the remaining cells. The translocation of transcription factors was analyzed: the nuclear area was subtracted from the whole cell area to obtain the cytoplasmic area. The transcription factor’s average fluorescence intensity was measured in the nuclear and cytoplasmic regions, and then the nuclear–cytoplasmic ratio was calculated.

### 2.6. Flow Cytometry

After 14 days of mouse bone marrow heterotypic culture exposure to static control and simulated microgravity conditions, flow cytometry was used to determine the ratio of hematopoietic and stromal cells. Pre-trypsinized associates were stained with PE-conjugated rat monoclonal antibodies against mouse CD45 (BD Biosciences, Franklin Lakes, NJ, USA) according to the manufacturer’s instructions. The proportion of CD45^+^ cells (hematopoietic compartment) and CD45− cells (stromal compartment) in the population was determined. The viability of the heterocellular culture was assessed by PI and Annexin V staining (Beckman Coulter, Indianapolis, IN, USA). The viability of the hematopoietic and stromal compartments separately was determined by co-staining with PE-conjugated CD45 antibodies and 7AAD (Beckman Coulter, Indianapolis, IN, USA). To evaluate fluorochrome unspecific staining, appropriate isotype controls were applied (Becton Dickinson (BD), Bergen, NJ, USA).

Cells were analyzed using a CytoFlex S flow cytometer (Beckman Coulter, Indianapolis, IN, USA). CytExpert software version 2.4 (Beckman Coulter, Indianapolis, IN, USA) was used for data collection and compensation. Further data processing and image acquisition were performed by Kaluza Analysis 2.1 (Beckman Coulter, Indianapolis, IN, USA).

### 2.7. Histochemical Evaluation of Osteogenic Potential

Osteopotential was determined by alkaline phosphatase (Alpl) activity using an alkaline phosphatase kit (Sigma-Aldrich, Burlington, MA, USA). Before staining, the medium was removed, and the cells were washed with PBS and fixed for 20 min in 4% paraformaldehyde, and then washed with PBS again.

Enzyme activity was determined by the presence of the formed stained product and the intensity of its staining on microphotographs using a microscope (Nikon Eclipse TiU, Tokyo, Japan). The intensity of the staining reflects enzyme activity. The digital images were then processed using Sigma Scan Pro 5 software (IBM Corporation, Armonk, NY, USA).

The mineralization of the extracellular matrix was determined by alizarin red S. Cells were fixed with 4% paraformaldehyde for 15 min and then washed several times. A 40 mM alizarin red S solution with 2.8% ammonium hydroxide was added for 2 min at room temperature and then removed, and the samples were gently washed with distilled water. Alizarin red S stains calcified matrix components red during the reaction. Random fields of view were photographed and processed using Sigma Scan Pro 5 software (IBM Corporation, Armonk, NY, USA) to quantify staining intensity.

### 2.8. Statistical Analysis

All experiments were performed in 3–5 independent settings. The results are presented as mean ± standard deviation (M ± SD) or as median ± interquartile range. The Kolmogorov–Smirnov test and the Shapiro–Wilk test were used to determine the normality of the samples. Statistically significant differences between experimental groups were assessed using the nonparametric Mann–Whitney test using GraphPad Prism 9.0 (GraphPad Software, La Jolla, CA, USA). The illustrations were created using GraphPad Prism 9.0 as well as the online graphics editor Biorender.com.

## 3. Results and Discussion

### 3.1. Characterization of the Heterotypic Bone Marrow Culture

Heterocellular associations consisting of fibroblast-like stromal cells and hematopoietic cells, mainly of a monocytic line, were used in in vitro experiments as a model of stromal and hematopoietic cell compartment interaction [36,42,43]. This approach allows us to maintain a cell microenvironment and biosignaling conditions that are close to in vivo conditions.

**Figure 2:**
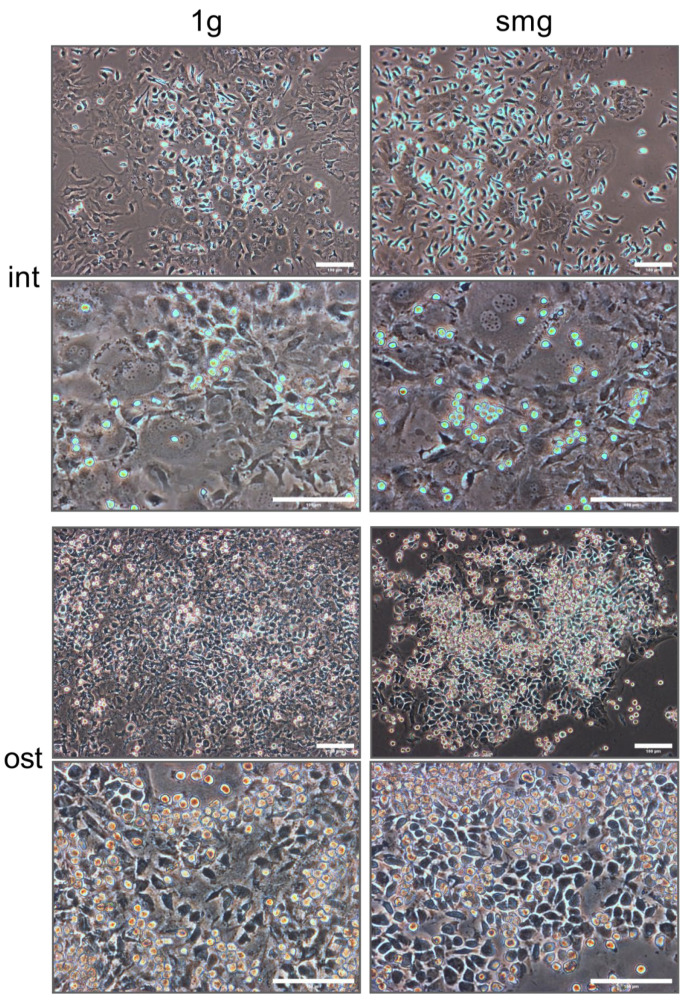
Heterotypic cell cultures of murine bone marrow after 14 days of in vitro exposure to 1 g (**left** column) or smg (**right** column). Top 4 images show intact cells, and bottom 4 images are osteoinduced cells. Phase contrast microscopy, scale bar—100 μm. Abbreviations: int (intact medium)—complete culture medium; ost—osteogenic culture medium; 1 g—standard culture conditions; smg—3D clinorotation for 14 days.

As the first step, we characterized two types of heterocellular bone marrow cultures: intact (int) and osteoinduced (ost). Both cultures were exposed to standard conditions (1 g) and simulated microgravity (smg) for 14 days. Following a 14-day cultivation period for the intact group, the cell morphology between 1 g and smg samples was found to be similar (Figure 2). In both groups, there were areas with larger cells that exhibited morphological features consistent with bone marrow stromal cells. A proportion of the hematopoietic cells, which were small, round and phase-light in morphology, was located on the surface of the larger stromal cells. A further proportion of the hematopoietic cells, which were phase-dull and displayed macrophage-like morphology, was located on the periphery of such clusters. The osteoinductors resulted in a higher density of heterocellular associate clusters compared to the control group (Figure 2). Furthermore, there was a notable increase in the density of both round, phase-light hematopoietic cells and phase-dull macrophage-like cells. The cell density was so high that the majority of stromal cells were covered by hematopoietic cells. The morphology of cells in the group with osteoinduction between 1 g and smg was also similar. In both the int and ost groups after smg, there were fewer heterocellular clusters and the density of cells within them was lower.

The heterocellular culture microphotographs were processed using CellProfiler 4 software. The total number of cells was counted. The number of cells in the intact group exposed to smg conditions decreased significantly by an average of 1.6 times compared to 1 g (1.15 ± 0.05 × 10^6^ cells/25 cm^2^—1 g, 0.7 ± 0.14 × 10^6^ cells/25 cm^2^—smg, *p* < 0.05, n = 3). In the osteoinduction group, there was an average 2.8-fold decrease in total cell count under smg conditions compared to 1 g (2.87 ± 0.54 × 10^6^ cells/25 cm^2^—1 g, 1.04 ± 0.21 × 10^6^ cells/25 cm^2^—smg, *p* < 0.05, n = 3). Therefore, it can be concluded that exposure to simulated microgravity resulted in a significant decrease in total cell count. Interestingly, the addition of osteoinductors did not reverse this effect. In fact, it resulted in a more pronounced decrease in the total number of cells.

**Figure 3:**
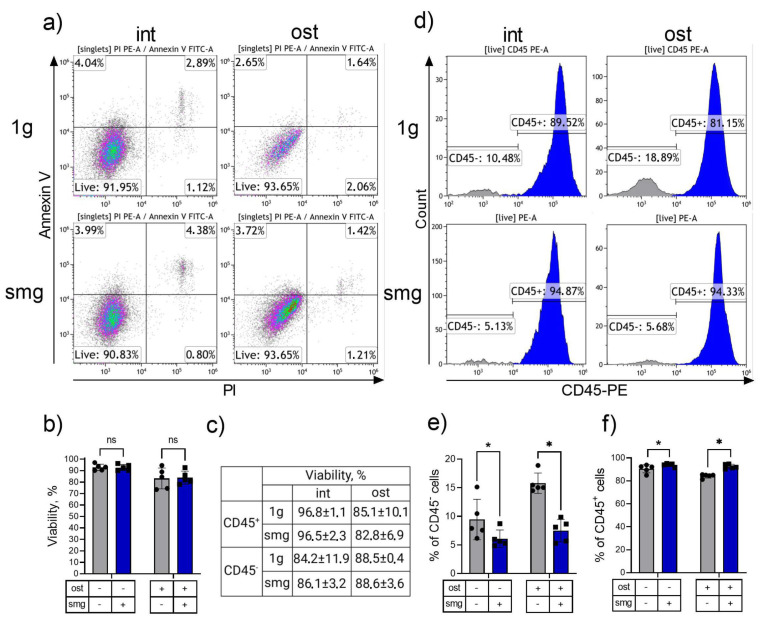
Flow cytometry analysis of intact and osteoinduced heterotypic cell culture from murine bone marrow under 1 g and smg conditions. Heterotypic cell culture viability analysis: (**a**) representative density plots of the heterotypic cell culture double-stained with PI and Annexin V; (**b**) the proportion of viable cells in the population, defined as PI-Annexin V-, n = 5, ns - not significant; (**c**) the proportion of viable cells in the CD45+ and CD45− populations, defined as 7AAD-negative cells, n = 5. Determination of the ratio of hematopoietic and stromal compartments in heterocellular culture: (**d**) representative histograms of CD45 staining showing hematopoietic cells (CD45+) and stromal cells (CD45−); (**e**) the proportion of stromal cells (CD45−), n = 5, *—*p* < 0.05; (**f**) the proportion of hematopoietic cells (CD45+), n = 5, *—*p* < 0.05. The data are presented as mean ± standard deviation (M ± SD); int (intact medium)—complete culture medium; ost—osteogenic culture medium; 1 g—standard culture conditions; smg—3D clinorotation for 14 days.

Analyses of the phenotype and viability of cultured bone marrow cells were conducted using flow cytometry. To analyze the viability of heterocellular cultures, the ratio of Annexin V- and PI-negative cells was determined (see Figure 3a,b). The viability of cells in both the intact and osteoinduction groups remained consistent under simulated microgravity conditions (Figure 3a,b). As the analyzed cultures are heterocellular, comprising hematopoietic cells expressing a pan-leukocyte antigen (CD45) as well as CD45-negative stromal cells, the next step was to assess the viability of each compartment separately. To this end, cell cultures were subjected to double staining with antibodies against CD45 conjugated to PE and 7AAD. The ratio of 7AAD-negative cells among the CD45+ hematopoietic cell population and the CD45− stromal cell population was determined. Viability was maintained in both populations under simulated microgravity, in both the intact and osteoinduced groups (Figure 3c).

The use of antibodies against CD45 enabled the separation of the hematopoietic (CD45+) and stromal (CD45−) cells (Figure 3d). As shown earlier in our work, stromal cells (CD45−) are represented mostly by committed cells with the CD45–Sca1– phenotype, a third of the population were progenitor cells with the CD45–Sca1+ phenotype, and a small proportion was represented by mesenchymal stem cells with the CD45–Sca1+CD29+CD105+ phenotype [36]. The ratio of stromal cells in the intact culture was found to decrease 1.5-fold on average under microgravity simulation (Figure 3e). Conversely, the osteoinduction group demonstrated a reduction in the proportion of stromal cells exceeding two-fold under smg conditions (Figure 3e).

The results demonstrated a notable decrease in the overall cell count within the heterocellular bone marrow culture following simulated microgravity. Immunophenotype analysis revealed a shift in the ratio of stromal and hematopoietic cells, with a greater proportion of hematopoietic cells (Figure 3f). The osteoinductors did not result in a correction of these changes. In fact, there were more pronounced effects on the total cell count and on the ratio of stromal cells.

It is possible that the effects observed are due to a decrease in the proliferative activity of the cells. Similar changes have been documented on numerous occasions after simulated microgravity. The 3D-clinorotation of human MSCs for 1 to 20 days has been shown to inhibit their proliferation [13,44,45,46]. A study by Dai et al. (2007) revealed that rat bone marrow mesenchymal stromal cells exposed to simulated microgravity exhibited inhibited proliferation and cell cycle arrest in the G0/G1 phase [10]. A reduction in the proliferation of mouse bone marrow-derived mesenchymal stem cells was observed following a 72 h microgravity simulation [47]. Furthermore, a 14-day period of simulated microgravity for a heterocellular culture of mouse bone marrow revealed a lack in the number of the most primitive MSCs (CD45-CD29+CD105+Sca1+) [36].

It is important to highlight that the initial and essential stage of osteodifferentiation is the proliferation of osteoprogenitor cells [48]. In light of the above, it seems reasonable to suggest that a decline in proliferation in microgravity may be a contributing factor to MSC osteodifferentiation inhibition.

A range of studies have demonstrated a reduction in the proliferative activity of hematopoietic cells. The total number and ratio of hematopoietic precursors and monocytic cells were reduced. The cell cycle slowed down. The expression of positive regulators of the cell cycle decreased, while the expression of negative regulators increased. The expression of hematopoietic factors that promote proliferation also decreased in both real and simulated microgravity [36,49,50,51,52,53,54,55,56,57].

### 3.2. Cell Osteopotential Analysis

It is known that exposure to microgravity causes MSCs and their committed progeny to preferentially differentiate into adipogenic lineage cells as opposed to osteogenic cells [6,12,44,58,59,60,61]. The osteopotential of adherent bone marrow cells under simulated microgravity was then assessed.

**Figure 4:**
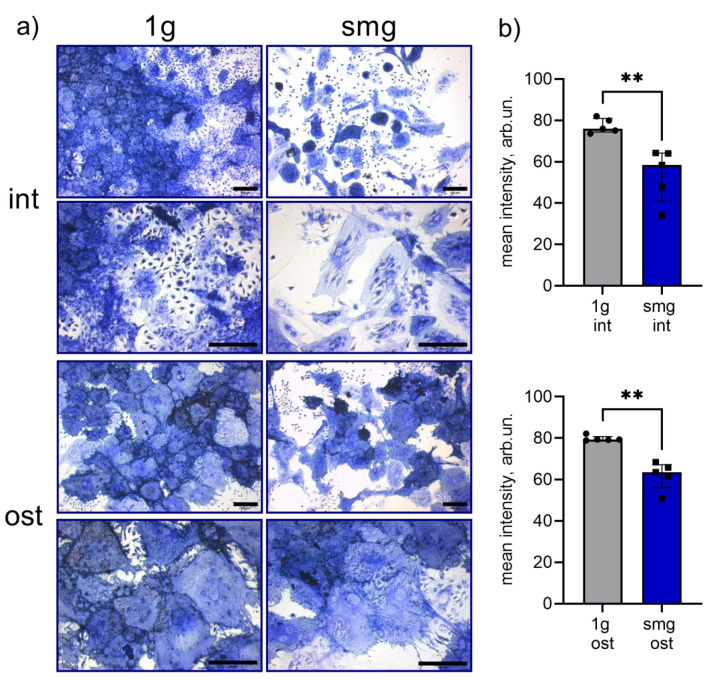
Histochemical evaluation of alkaline phosphatase activity in intact and osteoinduced heterotypic cell culture from murine bone marrow under 1 g and smg conditions. (**a**) Representative images, bright field microscopy, scale bar—100 μm. op 4 images are intact cells, and bottom 4 images are osteoinduced cells; (**b**) alkaline phosphatase activity assay by digital image processing, mean intensity, arb.un., n = 5, **—*p* < 0.01. The data are presented as mean ± standard deviation (M ± SD). Abbreviations: int (intact medium)—complete culture medium; ost—osteogenic culture medium; 1 g—standard culture conditions; smg—3D clinorotation for 14 days.

The activity of an early marker of osteodifferentiation, Alpl, was determined histochemically [62]. Alpl activity indicates that mesenchymal stromal cells differentiate into osteoblasts during osteogenesis [63]. Enzyme activity was determined by the presence and intensity of a colored product. In the intact group, simulated microgravity led to a decrease in Alpl activity by an average of 44% (Figure 4a,b). This may indicate a decrease in the osteopotential of bone marrow adherent cells. The addition of osteoinducers did not completely abolish this effect. There was a slight decrease in alkaline phosphatase activity by an average of 29% (Figure 4b).

**Figure 5:**
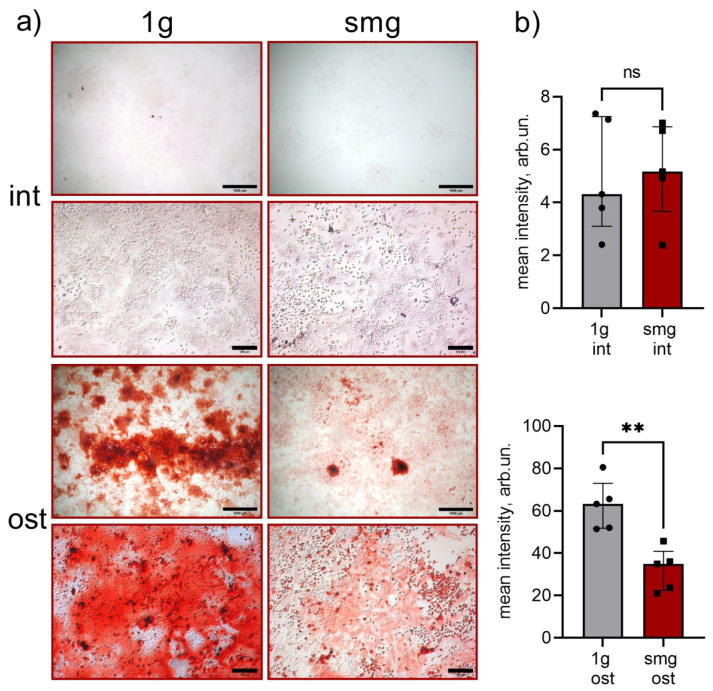
Mineralized matrix in intact and osteoinduced heterotypic cell culture from murine bone marrow under 1 g and smg conditions, alizarin red S staining. (**a**) Representative images, bright field microscopy, scale bar—1000 μm (top row), 100 μm (bottom row). Top 4 images are intact cells, and bottom 4 images are osteoinduced cells; (**b**) quantitative analysis of alizarin red S staining intensity by digital image processing, mean intensity, arb.un., n = 5, **—*p* < 0.01, ns - not significant. The data are presented as mean ± standard deviation (M ± SD). Abbreviations: int (intact medium)—complete culture medium; ost—osteogenic culture medium; 1 g—standard culture conditions; smg—3D clinorotation for 14 days.

The extracellular matrix (ECM) is important for osteogenic differentiation. It is secreted by osteoinduced MSCs and contains growth factors and many other proteins such as fibronectin, vitronectin, laminin, osteopontin and osteonectin [64]. Hydroxyapatite deposition is the final stage of osteoblast development [48]. The next step of our study was to assess extracellular matrix mineralization by alizarin red S staining, which labels calcium phosphate deposits [65,66]. In the intact group, no mineralized matrix formation was observed under either 1 g or smg exposure (Figure 5a,b). In the osteoinduction group, simulated microgravity caused a 1.9-fold decrease in ECM mineralization (Figure 5a,b).

It has been shown that intact, adherent murine bone marrow cultures exposed to simulated microgravity for 14 days show a decrease in Alpl activity, indicating a reduction in osteoprogenitor cell potential. This effect was maintained when the cell culture was exposed to constant levels of osteogenic inducers, as evidenced by the decrease in Alpl activity and matrix mineralization.

MSC osteogenic differentiation has several steps. MSCs differentiate into osteoblasts upon Runx2 activation, then mature, form a matrix and mineralize [67]. MSCs proliferate in the early stages of osteogenesis [48]. MSCs begin to express osteogenic markers such as alkaline phosphatase during early cell differentiation. Alpl expressed by osteoblasts is important for subsequent mineralization [62,68,69,70]. After a rising phase, Alpl levels decline and osteocalcin and osteopontin are secreted by late osteoblasts, followed by calcium and phosphate deposition during the mineralization phase [71,72,73].

Our findings align with existing data on the impact of microgravity on MSC osteopotential. Dai et al. (2007) found that 72 h of simulated microgravity inhibits the differentiation of rat bone marrow-derived mesenchymal stem cells (MSCs) toward osteoblasts [10]. Four-day periods of both real and simulated microgravity led to a slowdown in murine preosteoblast lineage MC3T3-E1 growth [74]. A nine-day period of microgravity reduced *ALP* gene expression in the human osteoblast line MG-63 [75]. The effects of real and simulated microgravity on osteoblast differentiation were found to be consistent. A seven-day period of simulated microgravity reduced the expression of osteoblastic markers such as Alpl and osteocalcin in human osteoblast-like cells [76]. Human bone marrow MSCs showed significant suppression of differentiation into osteoblasts against the background of osteogenic induction under a 7-day simulated microgravity condition [59]. The expression of Alpl, collagen 1, osteonectin and Runx2 was suppressed [59]. Zhang et al. showed that a 12-day period of real microgravity suppressed osteogenic differentiation of human bone marrow MSCs and led to adipogenic differentiation even under osteogenic induction conditions [61].

Furthermore, 48 h of simulated microgravity (on a 2D-clinostat) impedes osteodifferentiation of rat bone marrow MSCs [12]. Smg resulted in F-actin depolymerization and the inhibition of Taz nuclear translocation, a key effector of the Hippo signaling pathway essential for osteogenesis [12].

### 3.3. Paracrine Activity Evaluation

To maintain a balanced process of bone formation and bone resorption, various types of cells in the bone remodeling process, such as bone marrow mesenchymal stem cells (BM-MSCs), osteoblasts, osteoclasts and osteocytes, work together. Paracrine regulation is an essential part of bone remodeling.

In this regard, at the next stage, we assessed the content of pleiotropic cytokines such as IL-6, MCP-1 and GROβ, which are major components of the conditioned medium. In the intact group, simulated microgravity led to a 26% increase in the content of IL-6 and a 3.5-fold increase in the chemokine MCP-1 (Table 2). At the same time, exposing the heterotypic cell culture to osteogenic inducers not only completely compensated for this effect but also led to a 5.3-fold decrease in IL-6 and a 2.1-fold decrease in the chemokine GROβ under smg relative to 1 g (Table 2).

IL-6 plays an important role in maintaining the dynamic balance between bone formation and resorption [77,78]. The effect of autocrine/paracrine IL-6 on BM-MSC osteogenic differentiation is still controversial. BM-MSCs continuously secrete IL-6 upon osteogenic induction. With the progression of osteogenic differentiation, IL-6 production gradually increases and peaks on days 10 or 14 of induction, followed by a decrease in levels [79]. However, a large body of work indicates an inhibitory effect of IL-6 on osteoblast differentiation [31,80,81,82]. At the same time, IL-6 is considered a pro-osteoclastic factor that promotes osteoclast activation and differentiation through direct and indirect pathways [78,80,83]. Reduced trabecular and cortical bone and increased osteoclast activity are observed in mice that overexpress IL-6 [84]. It is known that IL-6 is involved in a number of age-related diseases, including osteoporosis [33,85].

According to numerous studies, chemokines influence bone remodeling through autocrine or paracrine mechanisms [86]. Monocyte chemoattractant protein-1 (MCP-1 or CCL2) is a member of the CC-motif chemokine family and is produced by various cell types such as monocytic, osteoprogenitor, endothelial, epithelial, smooth muscle, microglial, etc. [87,88]. CCL2 is a pro-osteoclastic cytokine that stimulates osteoclastogenesis [86]. CCL2 is one of the most highly induced genes in human osteoporosis [89].

CXCL2 (GROβ) attracts neutrophils during inflammation and is primarily produced by monocytes and macrophages. CXCL2 has been shown to stimulate osteoclast formation in vitro [90] and can also inhibit osteoblast differentiation [91].

Thus, the cytokine network plays a crucial role in bone remodeling. The dysregulation of cytokines can lead to bone diseases such as osteoporosis. It is well known that in real spaceflight and in ground-based microgravity simulation, the level of pro-inflammatory cytokines in peripheral blood and tissues increases [28,29,91,92]. In parallel with this, bone resorption and a slowdown in osteogenesis are shown [6]. Establishing the mechanisms of these changes is particularly important. The mechanosensitive Hippo signaling pathway plays a central role in cell functional activity regulation, including in proliferation and osteodifferentiation [8,93,94]. Establishing the role of Hippo signaling pathway components in the development of microgravity-associated osteopenia may potentially aid in the development of countermeasures. The importance of this is particularly emphasized by the fact that pro-inflammatory cytokines activate the Hippo signaling pathway [34]. This leads to Yap suppression, which is accompanied by a slowdown in BM-MSC osteodifferentiation [34,95].

Furthermore, it has been shown that after the knockdown of Yap1 in MC3T3-E1 cells, the expression of pro-inflammatory IL-6 is upregulated, but anti-inflammatory OPG is reduced [96]. Yap1 inhibits the induction of TNF-α-stimulated bone-resorbing mediators by suppressing the NF-κB signaling pathway in MC3T3-E1 cells [96]. It is known that the expression of pro-inflammatory cytokines (IL-6, TNF-α and MCP-1) stimulates NF-κB signaling, which inhibits osteoblast differentiation [97]. In our case, we have observed an upsurge in IL-6 and MCP-1 concentration under smg conditions. Moreover, the osteodifferentiation of stromal progenitors is reduced.

In addition to MSCs, osteoblasts and osteocytes, macrophages are also an important group that influences osteogenesis [98,99]. Activated M1 macrophages suppress MSC osteogenic differentiation by releasing various pro-inflammatory factors such as IL-1, IL-6 and TNF-α [100,101]. M1 macrophages also promote osteoclastogenesis [102]. Yap in macrophages upregulated the expression of classic pro-inflammatory cytokines including IL-6, TNF-α and MCP-1 by binding to their promoter regions through association with their Tead transcriptional factors [103].

Boro M. and others demonstrated that components of the Hippo signaling pathway, MST1 and MST2, control the secretion of GROβ. The MST1/2-dependent release of CXCL1 and CXCL2 by macrophages triggers paracrine signaling, which in turn leads to the production of innate immune responses such as beta-defensins, NOXs, iNOS and pro-inflammatory cytokines [104].

### 3.4. Transcriptomic Analysis of Genes Encoding Hippo Pathway Components

**Figure 6:**
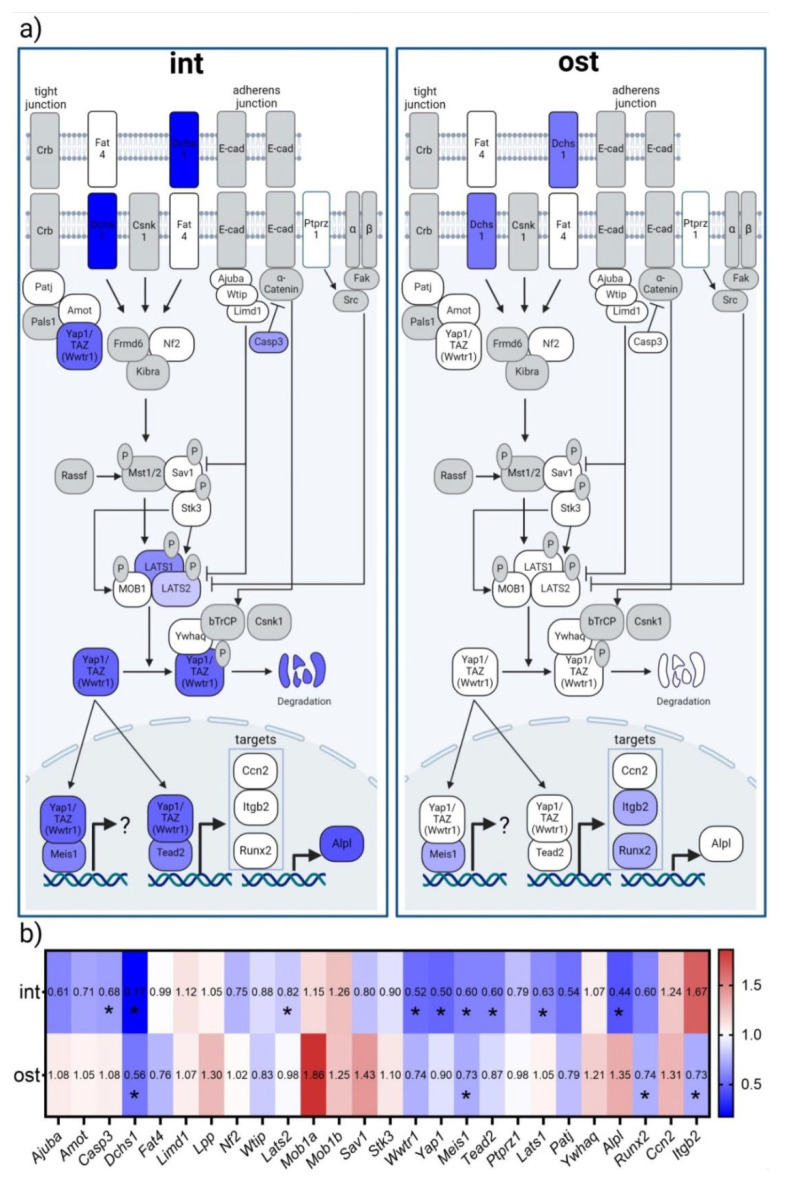
Simulated microgravity effects on transcriptomic profile of bone marrow cell culture. (**a**,**b**) Differential gene expression of Hippo signaling pathway components and regulators (*Ajuba*, *Amot*, *Casp3*, *Dchs1*, *Fat4*, *Limd1*, *Lpp*, *Nf2*, *Wtip*, *Lats2*, *Mob1a*, *Mob1b*, *Sav1*, *Stk3*, *Meis1*, *Ptprz1*, *Lats1*, *Patj*, *Ywhaq*), transcription factors Taz and Yap1 (*Wwtr1*, *Yap1*), their mediator Tead2 (*Tead2*) and Runx2 (*Runx2*), as well as target genes of the transcription factors Yap/Taz (*Ccn2*, *Itgb2*) and Runx2 (*Alpl*). (**b**) Heatmap, n = 3, *—*p* < 0.05. The data are presented as mean fold changes, smg vs. 1 g. Red—upregulation; blue—downregulation; white—no change; and gray—not assessed. Abbreviations: int (intact medium)—complete culture medium; ost—osteogenic culture medium; 1 g—standard culture conditions; smg—3D clinorotation for 14 days.

At the next stage, the expression of Hippo signaling pathway genes was analyzed in heterocellular associates. It was revealed that for the intact group, a 14-day period of simulated microgravity predominantly reduced the expression of genes which produce Hippo signaling pathway components (Figure 6). Among all the genes studied, the expression of the gene that encodes the intercellular adhesion molecule Dchs1, the genes of regulatory molecules Casp3, Lats1 and Lats2, and the gene of the transcription factor Meis1 were significantly downregulated. The expression of transcription factor genes Yap1, Taz (Wwtr1) and Tead2 decreased most significantly. At the same time, a significant decrease in the expression of the alkaline phosphatase gene Alpl is noted (Figure 6b).

Exposure to simulated microgravity against the background of osteogenic stimulation almost completely compensated for these effects. However, a significant downregulation of the genes that encode the intercellular adhesion molecule Dchs1, transcription factor gene Meis1 and the target gene of the Yap/Taz transcription factors—the integrin β-2 gene (Itgb2)—was noted. In addition, a decrease in the expression of an early marker of osteodifferentiation, gene Runx2, was observed (Figure 6b).

The Hippo pathway is known to coordinate cell proliferation, differentiation and death, thereby regulating overall cell number and final organ size [94]. It is regulated by multiple upstream signals including biophysical, biochemical, metabolic and stress signals, and its Yap/Taz effectors translate biophysical stimuli into gene expression to modulate cell proliferation, differentiation and survival.

The Hippo signaling pathway is represented as a kinase cascade in which Mst1/2 phosphorylates and activates Lats1/2, and Lats1/2 in turn phosphorylates and inactivates Yap/Taz [94]. The phosphorylation of Yap/Taz results in their cytoplasmic retention and proteasomal degradation. When the Hippo pathway is turned off, Yap/Taz translocate to the nucleus and interact with Tead1–4 to regulate gene expression [94].

Yes-associated protein (Yap) and transcriptional coactivator with PDZ-binding motif (Taz) are the major downstream effectors of the Hippo signaling pathway. Yap and Taz transduce a variety of ascending mechanical, structural and metabolic signals from the plasma membrane to the nucleus.

It is known that BM-MSC osteodifferentiation is regulated through the Hippo–Yap pathway [105,106,107]. Hippo plays an important role in the competitive adipose–osteogenic differentiation of MSCs. Yap promotes osteogenic differentiation and, conversely, antagonizes adipogenic differentiation [108].

In addition, increasing evidence points to the regulatory effects of Hippo–Yap/Taz on osteoclastogenesis and resorption activity, influencing bone homeostasis [21,93].

Yap/Taz are involved in the regulation of osteogenesis [21,22,23,24] and are the most important factors regulating the osteogenic differentiation of MSCs. The effect of positive regulation by Yap/Taz on the osteogenic differentiation of MSCs has been demonstrated in many studies [20,108,109,110]. Part of their effect may be achieved by regulating macrophage polarization [111]. Yap and Taz stimulate the osteogenic differentiation of MSCs by activating Runx2 [112,113,114]. Yap/Taz deletion has been shown to inhibit BM-MSC osteogenic differentiation [115,116]. RNAi-mediated deletion of Yap/Taz in BM-MSCs suppressed alkaline phosphatase activity and matrix mineralization even in an osteogenic environment [20,22]. According to the literature, Taz uniquely regulates the osteogenic differentiation of MSCs, while Yap plays a dual role [117], which is particularly noteworthy since its effects may differ depending on cross-talk with other signaling pathways [22,24,118,119].

In our work, we identified a predominantly decreased expression of Hippo signaling pathway component genes in the heterotypic murine bone marrow cell culture, including the main effectors of Yap1/Taz1 and the transcription factor Tead2 under simulated microgravity in vitro. This may be related to the pro-inflammatory activation shown in the previous step and may be the cause of the reduced osteopotential and cell proliferation under these conditions. Osteogenic induction partially compensated for this effect, but a downregulation of Yap1/Wwtr1 target genes *Itgb2* and *Runx2* was observed.

The observed changes are in good agreement with data from the literature. Yap translocation from the cytoplasm to the nucleus was impaired in glioblastoma cells and mesenchymal stem cells (MSCs) when the cells were cultured in RPM or a clinostat, respectively [120,121]. Using a 2D clinostat, a decrease in Taz activation and expression was shown due to F-actin depolymerization, which was the cause of reduced MSC osteogenic differentiation [12,122]. Decreased Yap expression has been demonstrated in human embryonic osteoblasts under real microgravity conditions [123].

### 3.5. Yap1 and Runx2 Nucleocytoplasmic Shuttling

Molecules that shuttle between the cytoplasm and the nucleus in response to mechanical forces, such as Yap and its ortholog Taz, are called molecular “transducers” of mechanical information. Yap/Taz have been shown to dynamically shuttle between the nucleus and cytoplasm, modulated by the Hippo pathway [124,125]. When Hippo signaling pathway activity is low, dephosphorylated, i.e., active, Yap/Taz translocate to the nucleus and interact with Tead1–4, modulating the expression of target genes [126]. In contrast, when the Hippo signaling pathway is activated, Yap/Taz are mainly restricted to the cytoplasm and Tead1–4 form a default repression complex with vestigial family member 4 (Vgll4) [127,128]. Therefore, Hippo signaling activation or inactivation may switch Tead1–4 from a repressor to an activator in gene transcription [94].

**Figure 7:**
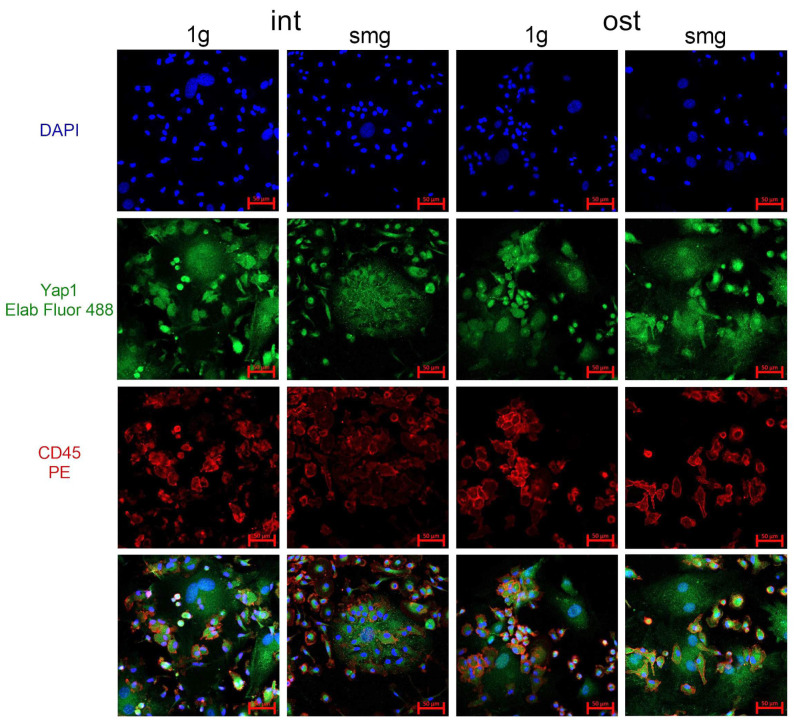
Confocal microscopy images of Yap1 in the intact and osteoinduced heterotypic cell cultures from murine bone marrow under 1 g and smg conditions. Representative confocal images of DAPI (blue)-, Yap1 (green)- and CD45 (red)-stained cell cultures. Scale bar—50 μm. Abbreviations: int (intact medium)—complete culture medium; ost—osteogenic culture medium; 1 g—standard culture conditions; smg—3D clinorotation for 14 days.

In this regard, in the next stage of this work, the nuclear–cytoplasmic distribution of Yap1 in a heterotypic mouse bone marrow culture was analyzed using confocal microscopy (Figure 7).

**Figure 8:**
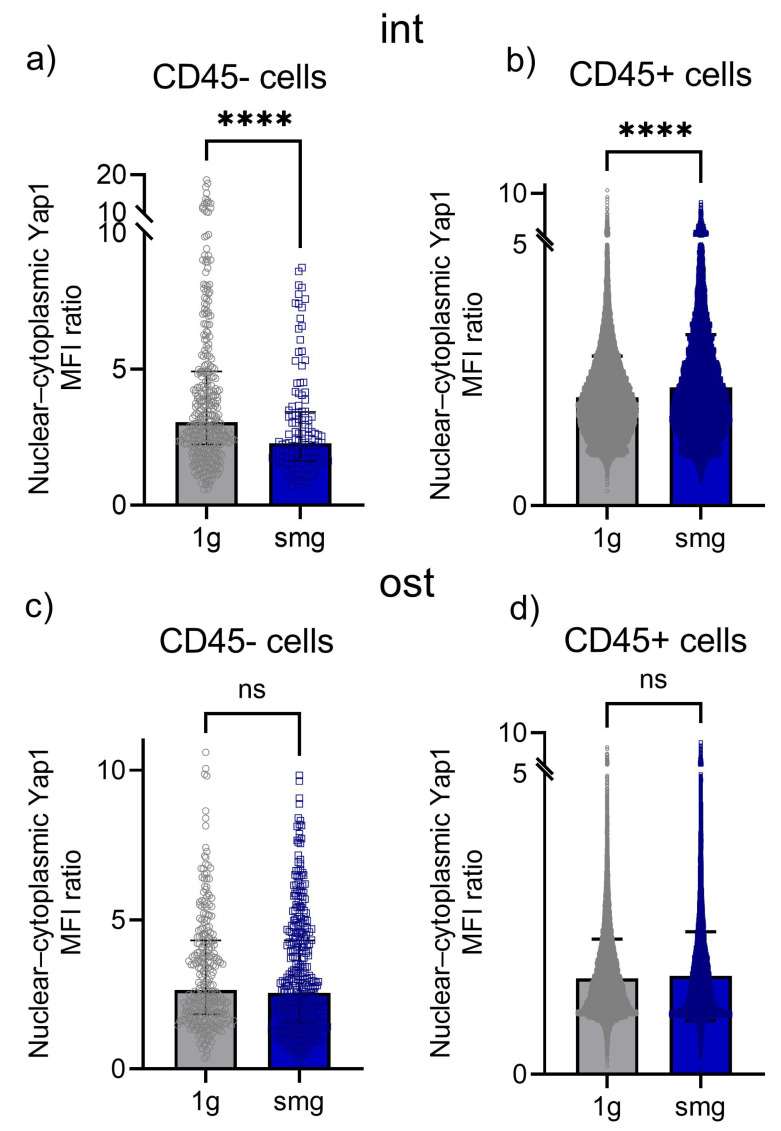
Yap1 translocation analysis in the intact and osteoinduced heterotypic cell cultures from murine bone marrow under 1 g and smg conditions. Quantitative analysis of confocal images. (**a**), (**b**)—The ratio of the mean fluorescence intensity (MFI) of nuclear and cytoplasmic Yap1 in the intact stromal cells (CD45−) and hematopoietic cells (CD45+), respectively. (**c**,**d**)—The ratio of the mean fluorescence intensity (MFI) of nuclear and cytoplasmic Yap1 in the osteoinduced cell culture containing stromal cells (CD45−) and hematopoietic cells (CD45+), respectively. The data from three independent experiments are presented as median ± interquartile range. ****—*p* < 0.0001, ns - not significant. Abbreviations: int (intact medium)—complete culture medium; ost—osteogenic culture medium; 1 g—standard culture conditions; smg—3D clinorotation for 14 days.

CD45 staining allowed us to determine the nuclear–cytoplasmic Yap1 MFI ratio separately in stromal (CD45−) and hematopoietic cells (CD45+). In total, in the intact group, 329 and 125 stromal cells under 1 g and smg conditions, respectively, as well as 42882 and 14,874 hematopoietic cells under 1 g and smg conditions, respectively, were analyzed from three independent experiments. Simulated microgravity resulted in a median 35% reduction in Yap1 nucleocytoplasmic distribution in stromal cells (Figure 8a). In hematopoietic cells, a weak but significant increase in the median of 6% in the nuclear–cytoplasmic distribution of Yap1 was observed (Figure 8b).

In the osteoinduction group, a total of 266 and 363 stromal cells under 1 g and smg conditions, respectively, and 22,315 and 21,452 hematopoietic cells under 1 g and smg conditions, respectively, were analyzed from three independent experiments. Under microgravity simulation, no changes in the nuclear–cytoplasmic distribution of Yap1 were observed in either stromal cells or hematopoietic cells against the background of osteoinduction (Figure 8c,d).

Runx2 was initially identified as a positive regulator of osteoblast differentiation [129]. It is one of the most studied transcription factors expressed in MSCs upon their commitment toward osteogenic differentiation [130,131]. Runx2 is required for the expression of multiple osteogenic genes, including collagen I, osteopontin, alkaline phosphatase, bone sialoprotein and osteocalcin [130]. Runx2 functions by binding to regulatory regions in osteogenic gene promoters to activate transcription. It was previously shown that the nuclear–cytoplasmic shuttling of Runx2 may modulate its transcriptional activity, as well as its ability to interact with other signal transduction pathways [132].

**Figure 9:**
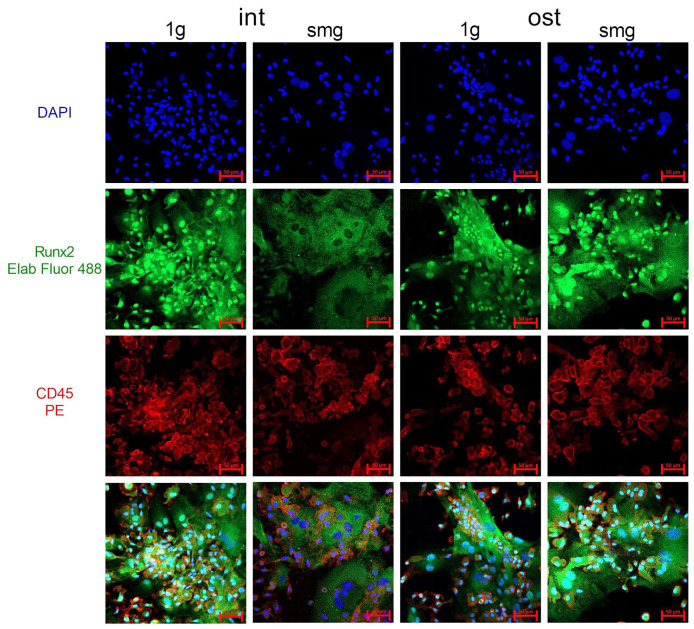
Confocal microscopy of Runx2 in the intact and osteoinduced heterotypic cell cultures from murine bone marrow under 1 g and smg conditions. Representative confocal images of DAPI (blue)-, Runx2 (green)- and CD45 (red)-stained cell cultures. Scale bar—50 μm. Abbreviations: int (intact medium)—complete culture medium; ost—osteogenic culture medium; 1 g—standard culture conditions; smg—3D clinorotation for 14 days.

The next step was to analyze the nuclear–cytoplasmic distribution of Runx2 in a heterotypic cell culture from mouse bone marrow using confocal microscopy (Figure 9).

**Figure 10:**
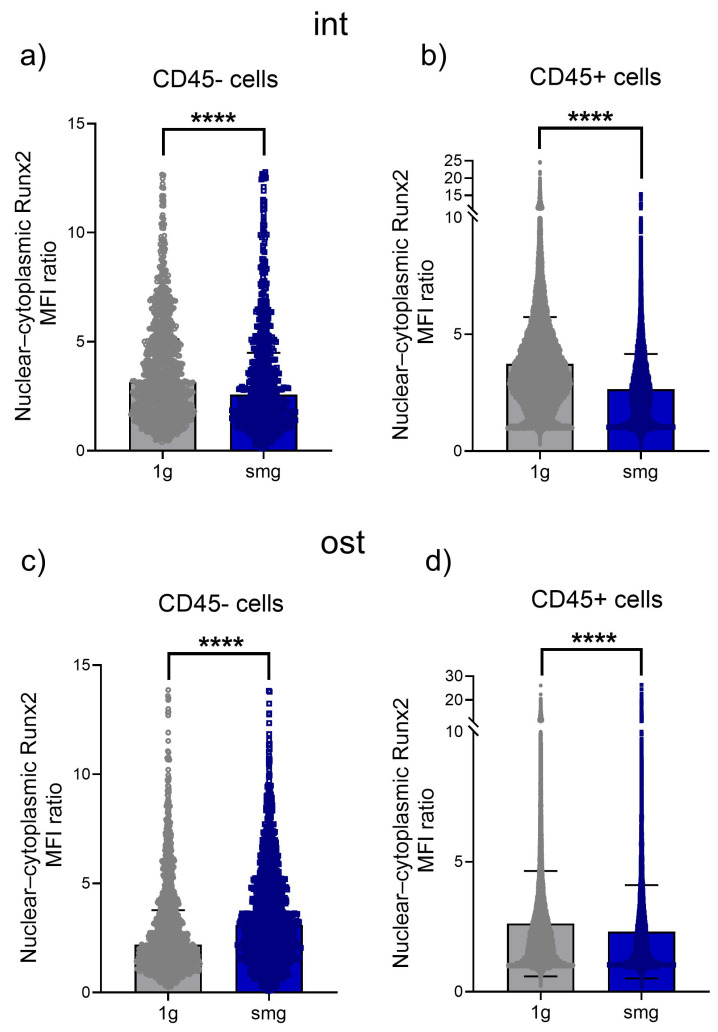
Analysis of Runx2 translocation in the intact and osteoinduced heterotypic cell cultures from mouse bone marrow under 1 g and smg conditions. Quantitative analysis of confocal images. (**a**,**b**)—The ratio of the mean fluorescence intensity (MFI) of nuclear and cytoplasmic Runx2 in the intact stromal cells (CD45−) and hematopoietic cells (CD45+), respectively. (**c**,**d**)—The ratio of the mean fluorescence intensity (MFI) of nuclear and cytoplasmic Runx2 in the osteoinduced cell culture containing stromal cells (CD45−) and hematopoietic cells (CD45+), respectively. The data from three independent experiments are presented as median ± interquartile range. ****—*p* < 0.0001. Abbreviations: int (intact medium)—complete culture medium; ost—osteogenic culture medium; 1 g—standard culture conditions; smg—3D clinorotation for 14 days.

In total, 949 and 707 stromal cells in the intact group under 1 g and smg conditions, respectively, and 49,236 and 17,050 hematopoietic cells under 1 g and smg conditions, respectively, were analyzed from three independent experiments. Simulated microgravity resulted in a median 22% reduction in Runx2 nuclear translocation in stromal cells (Figure 10a). In hematopoietic cells, the nuclear–cytoplasmic ratio decreased by 41% compared to 1 g (Figure 10b).

In the osteoinduction group, a total of 1387 and 1553 stromal cells under 1 g and smg conditions, respectively, and 47,497 and 12,443 hematopoietic cells under 1 g and smg conditions, respectively, were analyzed from three independent experiments. Under microgravity simulation, a significant increase in the median of 41% in the nuclear–cytoplasmic distribution of Runx2 was found in stromal cells compared to 1 g (Figure 10c). In hematopoietic cells, a 16% decrease in Runx2 translocation into the nucleus was observed compared to 1 g (Figure 10d).

Thus, a 14-day period of simulated microgravity induced a decrease in the nuclear translocation of Yap1 and the transcription factor Runx2, which may be a downstream target of Yap1, in stromal cells of the intact group. These changes may be the cause of the decrease in the proliferation and osteopotential of the analyzed cell culture. Exposure to osteogenic induction conditions not only compensated for the effect of a decreased nuclear–cytoplasmic distribution of Yap1 but also resulted in an increased nuclear entry of Runx2.

The impairment of Yap nuclear entry in murine BM-MSCs has previously been demonstrated under 72 h of simulated microgravity [120]. Moreover, the daily application of low-intensity vibration (LIVDT) in the presence of smg restored the smg-induced decrease in basal nuclear Yap to control levels and also increased lysophosphodiesterase (LPA)-induced nuclear Yap entry [120].

Under the simulated microgravity condition, in the hematopoietic compartment, the majority of which consists of monocytic cells, a slight increase in Yap1 nuclear translocation was detected upon exposure to the intact group, which was completely compensated by osteoinduction. At the same time, the nuclear–cytoplasmic distribution of Runx2 was reduced in the intact group. The osteoinductors only partially compensated for this effect.

The role of Runx2 in hematopoietic cells is largely unclear. However, it has been shown that the deletion of Runx2 in hematopoietic cells leads to a decrease in plasmacytoid dendritic cells that mediate type I interferon responses to viral infection [133]. The role of Yap1 in hematopoietic cells is less well known than in stromal cells. However, in addition to MSCs, osteoblasts and osteoclasts, macrophages play an important role in osteogenesis [102,134,135]. As key players in the osteoimmunological response, macrophages respond to a variety of biological signals to adapt to different microenvironments, including Hippo–Yap signaling. It has now been shown that Yap plays a complex role in the regulation of osteogenesis in part by regulating macrophage polarization depending on various stimuli [111]. Therefore, Yap is a potential therapeutic target to accelerate and improve bone defect repair. Although data are controversial, a number of studies indicate that Yap inhibition may mediate M2 macrophage polarization, thereby promoting osteogenesis [20,124,136,137,138]. Furthermore, indirect regulation of macrophage polarization may be mediated by Yap through cell–cell interactions. For example, the activation of Yap expression and Yap nuclear translocation enhances the paracrine function of MSCs, which in turn promotes the M2 polarization of surrounding macrophages, thereby suppressing inflammation and promoting osteogenesis [139].

Several studies indicate that Yap has a pro-inflammatory function in macrophages, and several inflammatory factors can promote Yap transcriptional activity in macrophages, forming a positive feedback loop in the inflammatory response [140]. In particular, macrophages overexpressing Yap showed an increase in the expression and production of the chemokine CCL2 [141]. In our work, simulated microgravity increased the content of MCP-1 (CCL2) in the intact heterotypic cell culture, which completely compensated for exposure to osteoinduction conditions.

## 4. Conclusions

By summarizing the obtained data, it can be concluded that simulated microgravity negatively affects stromal and hematopoietic cells when interacting in a heterotypic cell culture of murine bone marrow. This is evidenced by a decrease in cell proliferation and osteopotential. The change in the paracrine profile may be a reason for the lack of osteodifferentiation in the heterotypic bone marrow cell population. We suggest that the observed effects are associated with altered regulation of the Hippo signaling pathway during microgravity simulation.

The mechanosensitive signaling pathway Hippo, together with its main effector and mechanotransducer Yap, plays an important role in osteogenesis [21,22,23,24,105,106,107], a process that is negatively affected by microgravity [6]. A 14-day simulation of microgravity was accompanied by a predominant downregulation of genes that are components of the Hippo signaling pathway, including genes encoding transcription factors Yap1, Taz (Wwtr1) and Tead2, as well as the gene encoding an important component of osteodifferentiation, alkaline phosphatase Alpl. At the same time, a decrease in the translocation of Yap1 and Runx2 into the nucleus of stromal cells was revealed during microgravity simulation. Together, these changes may mediate impairments in osteodifferentiation and cell proliferation. In the hematopoietic compartment, a decrease in Runx2 nuclear–cytoplasmic shuttling was detected, while Yap1 nuclear translocation was slightly increased. The complex effects of Yap on osteogenesis are achieved through the regulation of both stromal and hematopoietic cells. Yap modulates the expression of downstream target genes through various signaling pathways in these cells, modulating their osteogenesis-related biological functions. Overall, we suggest that the complex consisting of these gravity-dependent changes may be one of the mechanisms of osteodegenerative phenomena that develop under long-term spaceflight.

Given the osteopenia associated with microgravity, finding effective countermeasures is a particularly important task. In this regard, studying the biochemistry of osteodifferentiation inducers may be an intriguing approach to preventing osteogenesis disorders. Exposing the heterotypic bone marrow cell culture to osteoinductors partially compensated for the negative effects of microgravity. Fewer changes in the transcriptional activity of Hippo signaling pathway component genes were observed, as well as the restoration of Yap1 nuclear–cytoplasmic shuttling and the increased translocation of Runx2 into the nucleus of stromal cells. This was accompanied by a decrease in the production of pro-inflammatory cytokines IL-6 and GROβ. Despite this positive effect, a significant decrease in proliferation and osteopotential remained in the cell culture, which may indicate the involvement of other mechanisms in the impairment of osteodifferentiation mediated by microgravity. In addition, the results obtained using osteoinductors may indicate multidirectional effects of Yap at several stages of osteodifferentiation, which is confirmed by the work of other researchers [24,142,143,144,145].

In conclusion, our of heterotypic bone marrow cell culture model, which allows for the maintenance of the close interaction between the stromal and hematopoietic compartments, confirmed the decrease in osteodifferentiation previously shown in real and simulated microgravity. Simulated microgravity caused inhibition of the osteogenic function of bone marrow stromal progenitors due to the downregulation of genes and the decrease in the nuclear translocation of Hippo signaling pathway effectors—YAP1 and it’s target RUNX2. The addition of osteoinducers partially compensated for the deficit in osteogenic function. The data obtained will be critical for understanding the effects of spaceflight on osteoprogenitor cell growth and differentiation via Hippo–Yap signaling.

## Figures and Tables

**Table 1:** The primer sequences used for qRT-PCR.

Target Gene		Primer Sequences
*Ajuba*	For	5′ AAATTACGCCCCGAAATGTG 3′
	Rev	5′ CTCACAGTGGTAGCACTCAA 3′
*Amot*	For	5′ TAGACCCCTGAATTACCCCT 3′
	Rev	5′ GATGGTGAGAGGCGAGATAG 3′
*Casp3*	For	5′ GCTTGGAACGGTACGCTAAG 3′
	Rev	5′ CACTGACTTGCTCCCATGTA 3′
*Dchs1*	For	5′ GCACCGCATCGAGGTC 3′
	Rev	5′ GCTCCACACCAGACTCTTAG 3′
*Fat4*	For	5′ TGGGCTGTGTAATGGAGTTT 3′
	Rev	5′ TGCCTTCCAATCTAGGACAC 3′
*Limd1*	For	5′ TTCGGTTCCTGTGTGAAATG 3′
	Rev	5′ TTCCTCTTAACTTCCTGCTGC 3′
*Lpp*	For	5′ TGTGCTGTCCTCAAGTCTTT 3′
	Rev	5′ ATCAAGCTCTCTCTCATGGC 3′
*Nf2*	For	5′ TCAAGAGATCACGCAACACT 3′
	Rev	5′ GGGTCATAGTCGCCATACTT 3′
*Wtip*	For	5′ TGGCATTTGTATCAAGTGTGG 3′
	Rev	5′ AGGAGTCACAGATGAAGCAG 3′
*Lats1*	For	5′ TCTGCTACACCAAAACCCAT 3′
	Rev	5′ GTCCGCACTCTTCTCACTAT 3′
*Lats2*	For	5′ TGTCGGGACACCAAATTACA 3′
	Rev	5′ CCAACCAGCATCTCAAAGAG 3′
*Mob1a*	For	5′ ACTGTTATTCTGCTGACCGA 3′
	Rev	5′ CAAACCTCATCACCAGGACT 3′
*Mob1b*	For	5′ CCGAGACCGTTCTCTACATT 3′
	Rev	5′ CGCATTTGTTTCCAGCATCA 3′
*Sav1*	For	5′ GAGTCACGGCTACATCTCTA 3′
	Rev	5′ ACTCCAGTGAGTGGTATTTGT 3′
*Stk3*	For	5′ GCGTCCAAGAGTAAGCTAAAA 3′
	Rev	5′ ACTTCCATAAGACCCTTCTCC 3′
*Wwtr1*	For	5′ CAGCAAGTCATCCACGTCAC 3′
	Rev	5′ GAAGGACTCCGGGAGGATCT 3′
*Yap1*	For	5′ TTTCGGCAGGCAATACGGAA 3′
	Rev	5′ GCATTCGGAGTCCCTCCATC 3′
*Meis1*	For	5′ TAATCTCCCTTCAGTGCAGC 3′
	Rev	5′ CCGCTTTCCTTGAATCAGTC 3′
*Tead2*	For	5′ ACGTCACAACCCGAAGATAA 3′
	Rev	5′ CCACTGCCTAGCTGAGATAA 3′
*Ywhaq*	For	5′ TTTTGAAGGCTTTTGCTGTTTC 3′
	Rev	5′ TGACCCATGAGGCTATCTGG 3′
*Patj*	For	5′ GGAAGATTTGCCTCTGTACCGAC 3′
	Rev	5′ GCTGAAGTTCGGTGTCTCCTCT 3′
*Ptprz1*	For	5′ GGAGTATCCAACAGTTCAGAGGC 3′
	Rev	5′ AAGTCAGGGCAGACACGATCAC 3′
*Runx2*	For	5′ CCTGAACTCTGCACCAAGTCCT 3′
	Rev	5′ TCATCTGGCTCAGATAGGAGGG 3′
*Hsp90AB1*	For	5′ CCTGAAGGTCATCCGCAAGAAC 3′
	Rev	5′ GGCGTCGGTTAGTGGAATCTTC 3′
*Alpl*	For	5′ CCAGAAAGACACCTTGACTGTGG 3′
	Rev	5′ TCTTGTCCGTGTCGCTCACCAT 3′
*Ccn2*	For	5′ TGCGAAGCTGACCTGGAGGAAA 3′
	Rev	5′ CCGCAGAACTTAGCCCTGTATG 3′
*Itgb2*	For	5′ CTTTCCGAGAGCAACATCCAGC 3′
	Rev	5′ GTTGCTGGAGTCGTCAGACAGT 3′

**Table 2:** Cytokine concentration in conditioned medium (pg/mL). The data are presented as mean ± standard deviation (M ± SD) (n = 3, *—*p* < 0.05).

	int		ost	
	1 g	smg	1 g	smg
IL-6	614.1 ± 98.7	778 ± 94.1 *	7472.4 ± 3936.5	1400.0 ± 650.9 *
MCP-1	90.6 ± 53.8	313.6 ± 57.7 *	3185.3 ± 43.2	3885.6 ± 25.6
GROβ	4291.8 ± 494	3653.3 ± 1019	22,542.7 ± 7847.9	10,814.9 ± 2755.3 *

Abbreviations: int (intact medium)—complete culture medium, ost—osteogenic culture medium. 1 g—standard culture conditions, smg—3D clinorotation for 14 days. Red—increase, blue—decrease in the smg group compared to 1g.

## Data Availability

The data presented in this study are available on request from the corresponding author. The data are not publicly available due to Institution policy.

## References

[1] Vernice N.A., Meydan C., Afshinnekoo E., Mason C.E. (2020). Long-Term Spaceflight and the Cardiovascular System. Precis. Clin. Med..

[2] Cao D., Song J., Ling S., Niu S., Lu L., Cui Z., Li Y., Hao S., Zhong G., Qi Z. (2019). Hematopoietic Stem Cells and Lineage Cells Undergo Dynamic Alterations under Microgravity and Recovery Conditions. FASEB J..

[3] Juhl O.J., Buettmann E.G., Friedman M.A., DeNapoli R.C., Hoppock G.A., Donahue H.J. (2021). Update on the Effects of Microgravity on the Musculoskeletal System. NPJ Microgravity.

[4] Gupta U., Baig S., Majid A., Bell S.M. (2023). The Neurology of Space Flight; How Does Space Flight Effect the Human Nervous System?. Life Sci. Space Res..

[5] Lansiaux E., Jain N., Yatin Chodnekar S., Siddiq A., Ibrahim M., Yèche M., Kantane I. (2024). Understanding the Complexities of Space Anaemia in Extended Space Missions: Revelations from Microgravitational Odyssey. Front. Physiol..

[6] Man J., Graham T., Squires-Donelly G., Laslett A.L. (2022). The Effects of Microgravity on Bone Structure and Function. NPJ Microgravity.

[7] LeBlanc A., Schneider V. (1992). Countermeasures against Space Flight Related Bone Loss. Acta Astronaut..

[8] Fu M., Hu Y., Lan T., Guan K.-L., Luo T., Luo M. (2022). The Hippo Signalling Pathway and Its Implications in Human Health and Diseases. Signal Transduct. Target. Ther..

[9] Blaber E.A., Dvorochkin N., Lee C., Alwood J.S., Yousuf R., Pianetta P., Globus R.K., Burns B.P., Almeida E.A. (2013). Microgravity Induces Pelvic Bone Loss through Osteoclastic Activity, Osteocytic Osteolysis, and Osteoblastic Cell Cycle Inhibition by CDKN1a/P21. PLoS ONE.

[10] Dai Z.Q., Wang R., Ling S.K., Wan Y.M., Li Y.H. (2007). Simulated Microgravity Inhibits the Proliferation and Osteogenesis of Rat Bone Marrow Mesenchymal Stem Cells. Cell Prolif..

[11] Markina E., Andreeva E., Andrianova I., Sotnezova E., Buravkova L. (2018). Stromal and Hematopoietic Progenitors from C57/BI/6N Murine Bone Marrow After 30-Day “BION-M1” Spaceflight. Stem Cells Dev..

[12] Chen Z., Luo Q., Lin C., Song G. (2015). Simulated Microgravity Inhibits Osteogenic Differentiation of Mesenchymal Stem Cells through down Regulating the Transcriptional Co-Activator TAZ. Biochem. Biophys. Res. Commun..

[13] Li L., Zhang C., Chen J.-L., Hong F.-F., Chen P., Wang J.-F. (2019). Effects of Simulated Microgravity on the Expression Profiles of RNA during Osteogenic Differentiation of Human Bone Marrow Mesenchymal Stem Cells. Cell Prolif..

[14] Jahed Z., Shams H., Mehrbod M., Mofrad M.R.K. (2014). Mechanotransduction Pathways Linking the Extracellular Matrix to the Nucleus. Int. Rev. Cell Mol. Biol..

[15] Goelzer M., Goelzer J., Ferguson M.l., Neu C.P., Uzer G. (2021). Nuclear Envelope Mechanobiology: Linking the Nuclear Structure and Function. Nucl. Austin Tex.

[16] Andreeva E., Matveeva D., Zhidkova O., Zhivodernikov I., Kotov O., Buravkova L. (2022). Real and Simulated Microgravity: Focus on Mammalian Extracellular Matrix. Life Basel Switz..

[17] Sun S.-Y., Zhang L.-Y., Chen X., Feng X.-Q. (2023). Biochemomechanical Tensegrity Model of Cytoskeletons. J. Mech. Phys. Solids.

[18] Gumbiner B.M., Kim N.G. (2014). The Hippo-YAP Signaling Pathway and Contact Inhibition of Growth. J. Cell Sci..

[19] Karaman R., Halder G. (2018). Cell Junctions in Hippo Signaling. Cold Spring Harb. Perspect. Biol..

[20] Dupont S., Morsut L., Aragona M., Enzo E., Giulitti S., Cordenonsi M., Zanconato F., Le Digabel J., Forcato M., Bicciato S. (2011). Role of YAP/TAZ in Mechanotransduction. Nature.

[21] Lee J., Youn B.U., Kim K., Kim J.H., Lee D.-H., Seong S., Kim I., Han S.-H., Che X., Choi J.-Y. (2015). Mst2 Controls Bone Homeostasis by Regulating Osteoclast and Osteoblast Differentiation. J. Bone Miner. Res. Off. J. Am. Soc. Bone Miner. Res..

[22] Park H.W., Kim Y.C., Yu B., Moroishi T., Mo J.-S., Plouffe S.W., Meng Z., Lin K.C., Yu F.-X., Alexander C.M. (2015). Alternative Wnt Signaling Activates YAP/TAZ. Cell.

[23] Xue X., Hong X., Li Z., Deng C.X., Fu J. (2017). Acoustic Tweezing Cytometry Enhances Osteogenesis of Human Mesenchymal Stem Cells through Cytoskeletal Contractility and YAP Activation. Biomaterials.

[24] Pan J.X., Xiong L., Zhao K., Zeng P., Wang B., Tang F.L., Sun D., Guo H.H., Yang X., Cui S. (2018). YAP Promotes Osteogenesis and Suppresses Adipogenic Differentiation by Regulating β-Catenin Signaling. Bone Res..

[25] Kim E., Riehl B.D., Bouzid T., Yang R., Duan B., Donahue H.J., Lim J.Y. (2024). YAP Mechanotransduction under Cyclic Mechanical Stretch Loading for Mesenchymal Stem Cell Osteogenesis Is Regulated by ROCK. Front. Bioeng. Biotechnol..

[26] Yagi R., Chen L., Shigesada K., Murakami Y., Ito Y. (1999). A WW Domain-containing Yes-associated Protein (YAP) Is a Novel Transcriptional Co-activator. EMBO J..

[27] Chuang L.S.H., Ito Y. (2021). The Multiple Interactions of RUNX with the Hippo–YAP Pathway. Cells.

[28] Mehta S.K., Crucian B.E., Stowe R.P., Simpson R.J., Ott C.M., Sams C.F., Pierson D.L. (2013). Reactivation of Latent Viruses Is Associated with Increased Plasma Cytokines in Astronauts. Cytokine.

[29] Crucian B., Zwart S., Mehta S., Stowe R., Uchakin P., Quiriarte H., Pierson D., Smith S.M., Sams C. (2013). Immune System Dysregulation Persists During Long-Duration Spaceflight. J. Allergy Clin. Immunol..

[30] Lacey D.C., Simmons P.J., Graves S.E., Hamilton J.A. (2009). Proinflammatory Cytokines Inhibit Osteogenic Differentiation from Stem Cells: Implications for Bone Repair during Inflammation. Osteoarthr. Cartil..

[31] Kaneshiro S., Ebina K., Shi K., Higuchi C., Hirao M., Okamoto M., Koizumi K., Morimoto T., Yoshikawa H., Hashimoto J. (2014). IL-6 Negatively Regulates Osteoblast Differentiation through the SHP2/MEK2 and SHP2/Akt2 Pathways in Vitro. J. Bone Miner. Metab..

[32] Du D., Zhou Z., Zhu L., Hu X., Lu J., Shi C., Chen F., Chen A. (2018). TNF-α Suppresses Osteogenic Differentiation of MSCs by Accelerating P2Y2 Receptor in Estrogen-Deficiency Induced Osteoporosis. Bone.

[33] Xu J., Yu L., Liu F., Wan L., Deng Z. (2023). The Effect of Cytokines on Osteoblasts and Osteoclasts in Bone Remodeling in Osteoporosis: A Review. Front. Immunol..

[34] Deng Y., Lu J., Li W., Wu A., Zhang X., Tong W., Ho K.K., Qin L., Song H., Mak K.K. (2018). Reciprocal Inhibition of YAP/TAZ and NF-κB Regulates Osteoarthritic Cartilage Degradation. Nat. Commun..

[35] Soleimani M., Nadri S. (2009). A Protocol for Isolation and Culture of Mesenchymal Stem Cells from Mouse Bone Marrow. Nat. Protoc..

[36] Markina E., Tyrina E., Ratushnyy A., Andreeva E., Buravkova L. (2023). Heterotypic Cell Culture from Mouse Bone Marrow under Simulated Microgravity: Lessons for Stromal Lineage Functions. Int. J. Mol. Sci..

[37] Borst A.G., Van Loon J.J.W.A. (2008). Technology and Developments for the Random Positioning Machine, RPM. Microgravity Sci. Technol..

[38] Wuest S.L., Stern P., Casartelli E., Egli M. (2017). Fluid Dynamics Appearing during Simulated Microgravity Using Random Positioning Machines. PLoS ONE.

[39] Livak K.J., Schmittgen T.D. (2001). Analysis of Relative Gene Expression Data Using Real-Time Quantitative PCR and the 2−ΔΔCT Method. Methods.

[40] Stirling D.R., Swain-Bowden M.J., Lucas A.M., Carpenter A.E., Cimini B.A., Goodman A. (2021). CellProfiler 4: Improvements in Speed, Utility and Usability. BMC Bioinform..

[41] Stringer C., Wang T., Michaelos M., Pachitariu M. (2021). Cellpose: A Generalist Algorithm for Cellular Segmentation. Nat. Methods.

[42] Reimann J., Burger H. (1979). In Vitro Proliferation of Haemopoietic Cells in the Presence of Adherent Cell Layers. I. Culture Conditions and Strain Dependence. Exp. Hematol..

[43] Bentley S.A., Foidart J.M. (1980). Some Properties of Marrow Derived Adherent Cells in Tissue Culture. Blood.

[44] Merzlikina N.V., Buravkova L.B., Romanov Y.A. (2004). The Primary Effects of Clinorotation on Cultured Human Mesenchymal Stem Cells. J. Gravitational Physiol..

[45] Gershovich J.G., Buravkova L.B. (2007). Morphofunctional Status and Osteogenic Differentiation Potential of Human Mesenchymal Stromal Precursor Cells during in Vitro Modeling of Microgravity Effects. Bull. Exp. Biol. Med..

[46] Chi Q.H.N., Son N.H., Chung C.D., Huan L.D., Diem H.T., Long L.T. (2020). Simulated Microgravity Reduces Proliferation and Reorganizes the Cytoskeleton of Human Umbilical Cord Mesenchymal Stem Cells. Physiol. Res..

[47] Touchstone H., Bryd R., Loisate S., Thompson M., Kim S., Puranam K., Senthilnathan A.N., Pu X., Beard R., Rubin J. (2019). Recovery of Stem Cell Proliferation by Low Intensity Vibration under Simulated Microgravity Requires LINC Complex. NPJ Microgravity.

[48] Quarles L.D., Yohay D.A., Lever L.W., Caton R., Wenstrup R.J. (1992). Distinct Proliferative and Differentiated Stages of Murine MC3T3-E1 Cells in Culture: An in Vitro Model of Osteoblast Development. J. Bone Miner. Res. Off. J. Am. Soc. Bone Miner. Res..

[49] Lammer C., Wagerer S., Saffrich R., Mertens D., Ansorge W., Hoffmann I. (1998). The cdc25B Phosphatase Is Essential for the G2/M Phase Transition in Human Cells. J. Cell Sci..

[50] Hatton J.P., Gaubert F., Lewis M.L., Darsel Y., Ohlmann P., Cazenave J.P., Schmitt D. (1999). The Kinetics of Translocation and Cellular Quantity of Protein Kinase C in Human Leukocytes Are Modified during Spaceflight. FASEB J..

[51] Plett P.A., Frankovitz S.M., Abonour R., Orschell-Traycoff C.M. (2001). Proliferation of human hematopoietic bone marrow cells in simulated microgravity. In Vitro Cell. Dev. Biol. Anim..

[52] Plett P.A., Abonour R., Frankovitz S.M., Orschell C.M. (2004). Impact of Modeled Microgravity on Migration, Differentiation, and Cell Cycle Control of Primitive Human Hematopoietic Progenitor Cells. Exp. Hematol..

[53] Maier J.A. (2006). Impact of Simulated Microgravity on Cell Cycle Control and Cytokine Release by U937 Cells. Int. J. Immunopathol. Pharmacol..

[54] Villa A., Versari S., Maier J.A., Bradamante S. (2005). Cell Behavior in Simulated Microgravity: A Comparison of Results Obtained with RWV and RPM. Gravitational Space Biol. Bull. Publ. Am. Soc. Gravitational Space Biol..

[55] Zheng L., Liu J.Z., Hu Y.W., Zhong T.Y., Xiong S.L., Wang W., Wang Q. (2011). Simulated Microgravity, Erythroid Differentiation, and the Expression of Transcription Factor GATA-1 in CD34+ Cells. Aviat. Space Environ. Med..

[56] Blaber E.A., Dvorochkin N., Torres M.L., Yousuf R., Burns B.P., Globus R.K., Almeida E.A.C. (2014). Mechanical Unloading of Bone in Microgravity Reduces Mesenchymal and Hematopoietic Stem Cell-Mediated Tissue Regeneration. Stem Cell Res..

[57] Wang P., Tian H., Zhang J., Qian J., Li L., Shi L., Zhao Y. (2019). Spaceflight/Microgravity Inhibits the Proliferation of Hematopoietic Stem Cells by Decreasing Kit-Ras/cAMP-CREB Pathway Networks as Evidenced by RNA-Seq Assays. FASEB J. Off. Publ. Fed. Am. Soc. Exp. Biol..

[58] Miyamoto A., Shigematsu T., Fukunaga T., Kawakami K., Mukai C., Sekiguchi C. (1998). Medical Baseline Data Collection on Bone and Muscle Change with Space Flight. Bone.

[59] Zayzafoon M., Gathings W.E., McDonald J.M. (2004). Modeled Microgravity Inhibits Osteogenic Differentiation of Human Mesenchymal Stem Cells and Increases Adipogenesis. Endocrinology.

[60] Ratushnyy A.Y., Buravkova L.B. (2017). Expression of Focal Adhesion Genes in Mesenchymal Stem Cells under Simulated Microgravity. Dokl. Biochem. Biophys..

[61] Zhang C., Li L., Jiang Y., Wang C., Geng B., Wang Y., Chen J., Liu F., Qiu P., Zhai G. (2018). Space Microgravity Drives Transdifferentiation of Human Bone Marrow-Derived Mesenchymal Stem Cells from Osteogenesis to Adipogenesis. FASEB J..

[62] Tenenbaum H.C. (1987). Levamisole and Inorganic Pyrophosphate Inhibit Beta-Glycerophosphate Induced Mineralization of Bone Formed in Vitro. Bone Miner..

[63] Capulli M., Paone R., Rucci N. (2014). Osteoblast and Osteocyte: Games without Frontiers. Arch. Biochem. Biophys..

[64] El-Amin S.F., Lu H.H., Khan Y., Burems J., Mitchell J., Tuan R.S., Laurencin C.T. (2003). Extracellular Matrix Production by Human Osteoblasts Cultured on Biodegradable Polymers Applicable for Tissue Engineering. Biomaterials.

[65] Proia A.D., Brinn N.T. (1985). Identification of Calcium Oxalate Crystals Using Alizarin Red S Stain. Arch. Pathol. Lab. Med..

[66] Friedman M.S., Long M.W., Hankenson K.D. (2006). Osteogenic Differentiation of Human Mesenchymal Stem Cells Is Regulated by Bone Morphogenetic Protein-6. J. Cell. Biochem..

[67] Liu Z., Wang Q., Zhang J., Qi S., Duan Y., Li C. (2023). The Mechanotransduction Signaling Pathways in the Regulation of Osteogenesis. Int. J. Mol. Sci..

[68] Wennberg C., Hessle L., Lundberg P., Mauro S., Narisawa S., Lerner U.H., Millán J.L. (2000). Functional Characterization of Osteoblasts and Osteoclasts from Alkaline Phosphatase Knockout Mice. J. Bone Miner. Res. Off. J. Am. Soc. Bone Miner. Res..

[69] Golub E.E., Boesze-Battaglia K. (2007). The Role of Alkaline Phosphatase in Mineralization. Curr. Opin. Orthop..

[70] Boonrungsiman S., Gentleman E., Carzaniga R., Evans N.D., McComb D.W., Porter A.E., Stevens M.M. (2012). The Role of Intracellular Calcium Phosphate in Osteoblast-Mediated Bone Apatite Formation. Proc. Natl. Acad. Sci. USA.

[71] Huang W., Yang S., Shao J., Li Y.P. (2007). Signaling and Transcriptional Regulation in Osteoblast Commitment and Differentiation. Front. Biosci. J. Virtual Libr..

[72] Hoemann C.D., El-Gabalawy H., McKee M.D. (2009). In Vitro Osteogenesis Assays: Influence of the Primary Cell Source on Alkaline Phosphatase Activity and Mineralization. Pathol. Biol..

[73] Zhu S., Chen W., Masson A., Li Y.P. (2024). Cell Signaling and Transcriptional Regulation of Osteoblast Lineage Commitment, Differentiation, Bone Formation, and Homeostasis. Cell Discov..

[74] Hughes-Fulford M., Lewis M.I. (1996). Effects of Microgravity on Osteoblast Growth Activation. Exp. Cell Res..

[75] Carmeliet G., Nys G., Bouillon R. (1997). Microgravity Reduces the Differentiation of Human Osteoblastic MG-63 Cells. J. Bone Miner. Res..

[76] Kunisada T., Kawai A., Inoue H., Namba M. (1997). Effects of Simulated Microgravity on Human Osteoblast-like Cells in Culture. Acta Med. Okayama.

[77] Scheller J., Chalaris A., Schmidt-Arras D., Rose-John S. (2011). The Pro- and Anti-Inflammatory Properties of the Cytokine Interleukin-6. Biochim. Biophys. Acta.

[78] Wu Q., Zhou X., Huang D., Ji Y., Kang F. (2017). IL-6 Enhances Osteocyte-Mediated Osteoclastogenesis by Promoting JAK2 and RANKL Activity In Vitro. Cell. Physiol. Biochem. Int. J. Exp. Cell. Physiol. Biochem. Pharmacol..

[79] Xie Z., Tang S., Ye G., Wang P., Li J., Liu W., Li M., Wang S., Wu X., Cen S. (2018). Interleukin-6/Interleukin-6 Receptor Complex Promotes Osteogenic Differentiation of Bone Marrow-Derived Mesenchymal Stem Cells. Stem Cell Res. Ther..

[80] Kudo O., Sabokbar A., Pocock A., Itonaga I., Fujikawa Y., Athanasou N.A. (2003). Interleukin-6 and Interleukin-11 Support Human Osteoclast Formation by a RANKL-Independent Mechanism. Bone.

[81] Feng W., Liu H., Luo T., Liu D., Du J., Sun J., Wang W., Han X., Yang K., Guo J. (2022). Author Correction: Combination of IL-6 and sIL-6R Differentially Regulate Varying Levels of RANKL-Induced Osteoclastogenesis through NF-κB, ERK and JNK Signaling Pathways. Sci. Rep..

[82] Li X., Zhou Z.Y., Zhang Y.Y., Yang H.I. (2016). IL-6 Contributes to the Defective Osteogenesis of Bone Marrow Stromal Cells from the Vertebral Body of the Glucocorticoid-Induced Osteoporotic Mouse. PLoS ONE.

[83] Palmqvist P., Persson E., Conaway H.H., Lerner U.H. (2002). IL-6, Leukemia Inhibitory Factor, and Oncostatin M Stimulate Bone Resorption and Regulate the Expression of Receptor Activator of NF-Kappa B Ligand, Osteoprotegerin, and Receptor Activator of NF-Kappa B in Mouse Calvariae. J. Immunol..

[84] De Benedetti F., Rucci N., Del Fattore A., Peruzzi B., Paro R., Longo M., Vivarelli M., Muratori F., Berni S., Ballanti P. (2006). Impaired Skeletal Development in Interleukin-6-Transgenic Mice: A Model for the Impact of Chronic Inflammation on the Growing Skeletal System. Arthritis Rheum..

[85] Koshihara Y., Suematsu A., Feng D., Okawara R., Ishibashi H., Yamamoto S. (2002). Osteoclastogenic Potential of Bone Marrow Cells Increases with Age in Elderly Women with Fracture. Mech. Ageing Dev..

[86] Brylka L.J., Schinke T. (2019). Chemokines in Physiological and Pathological Bone Remodeling. Front. Immunol..

[87] Deshmane S.L., Kremlev S., Amini S., Sawaya B.E. (2009). Monocyte Chemoattractant Protein-1 (MCP-1): An Overview. J. Interferon Cytokine Res. Off. J. Int. Soc. Interferon Cytokine Res..

[88] Qian B.Z., Li J., Zhang H., Kitamura T., Zhang J., Campion L.R., Kaiser E.A., Snyder L.A., Pollard J.W. (2011). CCL2 Recruits Inflammatory Monocytes to Facilitate Breast-Tumour Metastasis. Nature.

[89] Hopwood B., Tsykin A., Findlay D.M., Fazzalari N.I. (2009). Gene Expression Profile of the Bone Microenvironment in Human Fragility Fracture Bone. Bone.

[90] Hardaway A.L., Herroon M.K., Rajagurubandara E., Podgorski I. (2015). Marrow Adipocyte-Derived CXCL1 and CXCL2 Contribute to Osteolysis in Metastatic Prostate Cancer. Clin. Exp. Metastasis.

[91] Yang Y., Zhou X., Li Y., Chen A., Liang W., Liang G., Huang B., Li Q., Jin D. (2019). CXCL2 Attenuates Osteoblast Differentiation by Inhibiting the ERK1/2 Signaling Pathway. J. Cell Sci..

[92] He B., Yin X., Hao D., Zhang X., Zhang Z., Zhang K., Yang X. (2020). Blockade of IL-6 Alleviates Bone Loss Induced by Modeled Microgravity in Mice. Can. J. Physiol. Pharmacol..

[93] Yang W., Han W., Qin A., Wang Z., Xu J., Qian Y. (2018). The Emerging Role of Hippo Signaling Pathway in Regulating Osteoclast Formation. J. Cell. Physiol..

[94] Zhong Z., Jiao Z., Yu F.-X. (2024). The Hippo Signaling Pathway in Development and Regeneration. Cell Rep..

[95] Huang F., Wei G., Wang H., Zhang Y., Lan W., Xie Y., Wu G. (2024). Fibroblasts Inhibit Osteogenesis by Regulating Nuclear-Cytoplasmic Shuttling of YAP in Mesenchymal Stem Cells and Secreting DKK1. Biol. Res..

[96] Yang B., Sun H., Xu X., Zhong H., Wu Y., Wang J. (2020). YAP1 Inhibits the Induction of TNF-α-Stimulated Bone-Resorbing Mediators by Suppressing the NF-κB Signaling Pathway in MC3T3-E1 Cells. J. Cell. Physiol..

[97] Chang J., Wang Z., Tang E., Fan Z., McCauley L., Franceschi R., Guan K., Krebsbach P.H., Wang C.-Y. (2009). Inhibition of Osteoblastic Bone Formation by Nuclear Factor-kappaB. Nat. Med..

[98] Gruber R. (2019). Osteoimmunology: Inflammatory Osteolysis and Regeneration of the Alveolar Bone. J. Clin. Periodontol..

[99] Tsukasaki M., Takayanagi H. (2019). Osteoimmunology: Evolving Concepts in Bone-Immune Interactions in Health and Disease. Nat. Rev. Immunol..

[100] Pajarinen J., Lin T., Gibon E., Kohno Y., Maruyama M., Nathan K., Lu L., Yao Z., Goodman S.B. (2019). Mesenchymal Stem Cell-Macrophage Crosstalk and Bone Healing. Biomaterials.

[101] Kushioka J., Chow S.K.-H., Toya M., Tsubosaka M., Shen H., Gao Q., Li X., Zhang N., Goodman S.B. (2023). Bone Regeneration in Inflammation with Aging and Cell-Based Immunomodulatory Therapy. Inflamm. Regen..

[102] Schlundt C., El Khassawna T., Serra A., Dienelt A., Wendler S., Schell H., van Rooijen N., Radbruch A., Lucius R., Hartmann S. (2018). Macrophages in Bone Fracture Healing: Their Essential Role in Endochondral Ossification. Bone.

[103] Song K., Kwon H., Han C., Chen W., Zhang J., Ma W., Dash S., Gandhi C.R., Wu T. (2020). Yes-Associated Protein in Kupffer Cells Enhances the Production of Proinflammatory Cytokines and Promotes the Development of Nonalcoholic Steatohepatitis. Hepatol. Baltim. Md.

[104] Boro M., Singh V., Balaji K.N. (2016). Mycobacterium Tuberculosis-Triggered Hippo Pathway Orchestrates CXCL1/2 Expression to Modulate Host Immune Responses. Sci. Rep..

[105] Murakami K., Kikugawa S., Kobayashi Y., Uehara S., Suzuki T., Kato H., Udagawa N., Nakamura Y. (2018). Olfactomedin-like Protein OLFML1 Inhibits Hippo Signaling and Mineralization in Osteoblasts. Biochem. Biophys. Res. Commun..

[106] An J., Li G., Zhang J., Zhou H., Jiang J., Wang X., Feng X., Wang S. (2019). GNAS Knockdown Suppresses Osteogenic Differentiation of Mesenchymal Stem Cells via Activation of Hippo Signaling Pathway. J. Cell. Physiol..

[107] Brandão A.S., Bensimon-Brito A., Lourenço R., Borbinha J., Soares A.R., Mateus R., Jacinto A. (2019). Yap Induces Osteoblast Differentiation by Modulating Bmp Signalling during Zebrafish Caudal Fin Regeneration. J. Cell Sci..

[108] Lorthongpanich C., Thumanu K., Tangkiettrakul K., Jiamvoraphong N., Laowtammathron C., Damkham N., U-Pratya Y., Issaragrisil S. (2019). YAP as a Key Regulator of Adipo-Osteogenic Differentiation in Human MSCs. Stem Cell Res. Ther..

[109] Hong J.-H., Hwang E.S., McManus M.T., Amsterdam A., Tian Y., Kalmukova R., Mueller E., Benjamin T., Spiegelman B.M., Sharp P.A. (2005). TAZ, a Transcriptional Modulator of Mesenchymal Stem Cell Differentiation. Science.

[110] Zhang Q., Guo Y., Yu H., Tang Y., Yuan Y., Jiang Y., Chen H., Gong P., Xiang L. (2019). Receptor Activity-Modifying Protein 1 Regulates the Phenotypic Expression of BMSCs via the Hippo/Yap Pathway. J. Cell. Physiol..

[111] Wang H., Yu H., Huang T., Wang B., Xiang L. (2023). Hippo-YAP/TAZ Signaling in Osteogenesis and Macrophage Polarization: Therapeutic Implications in Bone Defect Repair. Genes Dis..

[112] Matsumoto Y., La Rose J., Kent O.A., Wagner M.J., Narimatsu M., Levy A.D., Omar M.H., Tong J., Krieger J.R., Riggs E. (2016). Reciprocal Stabilization of ABL and TAZ Regulates Osteoblastogenesis through Transcription Factor RUNX2. J. Clin. Investig..

[113] Park J.S., Kim M., Song N.J., Kim J.H., Seo D., Lee J., Jung S.M., Lee J.Y., Lee J., Lee Y.S. (2019). A Reciprocal Role of the Smad4-Taz Axis in Osteogenesis and Adipogenesis of Mesenchymal Stem Cells. Stem Cells Dayt. Ohio.

[114] Lin X., Yang H., Wang L., Li W., Diao S., Du J., Wang S., Dong R., Li J., Fan Z. (2019). AP2a Enhanced the Osteogenic Differentiation of Mesenchymal Stem Cells by Inhibiting the Formation of YAP/RUNX2 Complex and BARX1 Transcription. Cell Prolif..

[115] Tang Y., Feinberg T., Keller E.T., Li X.Y., Weiss S.J. (2016). Snail/Slug Binding Interactions with YAP/TAZ Control Skeletal Stem Cell Self-Renewal and Differentiation. Nat. Cell Biol..

[116] Kegelman C.D., Mason D.E., Dawahare J.H., Horan D.J., Vigil G.D., Howard S.S., Robling A.G., Bellido T.M., Boerckel J.D. (2018). Skeletal Cell YAP and TAZ Combinatorially Promote Bone Development. FASEB J. Off. Publ. Fed. Am. Soc. Exp. Biol..

[117] Kegelman C.D., Coulombe J.C., Jordan K.M., Horan D.J., Qin L., Robling A.G., Ferguson V.I., Bellido T.M., Boerckel J.D. (2020). YAP and TAZ Mediate Osteocyte Perilacunar/Canalicular Remodeling. J. Bone Miner. Res. Off. J. Am. Soc. Bone Miner. Res..

[118] Seo E., Basu-Roy U., Gunaratne P.H., Coarfa C., Lim D.S., Basilico C., Mansukhani A. (2013). SOX2 Regulates YAP1 to Maintain Stemness and Determine Cell Fate in the Osteo-Adipo Lineage. Cell Rep..

[119] Seo J., Kim J. (2018). Regulation of Hippo Signaling by Actin Remodeling. BMB Rep..

[120] Thompson M., Woods K., Newberg J., Oxford J.T., Uzer G. (2020). Low-Intensity Vibration Restores Nuclear YAP Levels and Acute YAP Nuclear Shuttling in Mesenchymal Stem Cells Subjected to Simulated Microgravity. NPJ Microgravity.

[121] Silvani G., Bradbury P., Basirun C., Mehner C., Zalli D., Poole K., Chou J. (2022). Testing 3D Printed Biological Platform for Advancing Simulated Microgravity and Space Mechanobiology Research. NPJ Microgravity.

[122] Chen Z., Luo Q., Lin C., Kuang D., Song G. (2016). Simulated Microgravity Inhibits Osteogenic Differentiation of Mesenchymal Stem Cells via Depolymerizing F-Actin to Impede TAZ Nuclear Translocation. Sci. Rep..

[123] Wubshet N.H., Cai G., Chen S.J., Sullivan M., Reeves M., Mays D., Harrison M., Varnado P., Yang B., Arreguin-Martinez E. (2024). Cellular Mechanotransduction of Human Osteoblasts in Microgravity. NPJ Microgravity.

[124] Elosegui-Artola A., Andreu I., Beedle A.E.M., Lezamiz A., Uroz M., Kosmalska A.J., Oria R., Kechagia J.Z., Rico-Lastres P., Le Roux A.-L. (2017). Force Triggers YAP Nuclear Entry by Regulating Transport across Nuclear Pores. Cell.

[125] Manning S.A., Kroeger B., Harvey K.F. (2020). The Regulation of Yorkie, YAP and TAZ: New Insights into the Hippo Pathway. Dev. Camb. Engl..

[126] Zhao B., Ye X., Yu J., Li L., Li W., Li S., Yu J., Lin J.D., Wang C.-Y., Chinnaiyan A.M. (2008). TEAD Mediates YAP-Dependent Gene Induction and Growth Control. Genes Dev..

[127] Guo T., Lu Y., Li P., Yin M.-X., Lv D., Zhang W., Wang H., Zhou Z., Ji H., Zhao Y. (2013). A Novel Partner of Scalloped Regulates Hippo Signaling via Antagonizing Scalloped-Yorkie Activity. Cell Res..

[128] Koontz L.M., Liu-Chittenden Y., Yin F., Zheng Y., Yu J., Huang B., Chen Q., Wu S., Pan D. (2013). The Hippo Effector Yorkie Controls Normal Tissue Growth by Antagonizing Scalloped-Mediated Default Repression. Dev. Cell.

[129] Ducy P., Zhang R., Geoffroy V., Ridall A.L., Karsenty G. (1997). Osf2/Cbfa1: A Transcriptional Activator of Osteoblast Differentiation. Cell.

[130] Ducy P. (2000). Cbfa1: A Molecular Switch in Osteoblast Biology. Dev. Dyn. Off. Publ. Am. Assoc. Anat..

[131] Yamaguchi A., Komori T., Suda T. (2000). Regulation of Osteoblast Differentiation Mediated by Bone Morphogenetic Proteins, Hedgehogs, and Cbfa1. Endocr. Rev..

[132] Pockwinse S.M., Rajgopal A., Young D.W., Mujeeb K.A., Nickerson J., Javed A., Redick S., Lian J.B., van Wijnen A.J., Stein J.I. (2006). Microtubule-Dependent Nuclear-Cytoplasmic Shuttling of Runx2. J. Cell. Physiol..

[133] Sawai C.M., Sisirak V., Ghosh H.S., Hou E.Z., Ceribelli M., Staudt L.M., Reizis B. (2013). Transcription Factor Runx2 Controls the Development and Migration of Plasmacytoid Dendritic Cells. J. Exp. Med..

[134] Chang M.K., Raggatt L.-J., Alexander K.A., Kuliwaba J.S., Fazzalari N.L., Schroder K., Maylin E.R., Ripoll V.M., Hume D.A., Pettit A.R. (2008). Osteal Tissue Macrophages Are Intercalated throughout Human and Mouse Bone Lining Tissues and Regulate Osteoblast Function in Vitro and in Vivo. J. Immunol. Baltim..

[135] Vi L., Baht G.S., Whetstone H., Ng A., Wei Q., Poon R., Mylvaganam S., Grynpas M., Alman B.A. (2015). Macrophages Promote Osteoblastic Differentiation In-Vivo: Implications in Fracture Repair and Bone Homeostasis. J. Bone Miner. Res. Off. J. Am. Soc. Bone Miner. Res..

[136] Zhou X., Li W., Wang S., Zhang P., Wang Q., Xiao J., Zhang C., Zheng X., Xu X., Xue S. (2019). YAP Aggravates Inflammatory Bowel Disease by Regulating M1/M2 Macrophage Polarization and Gut Microbial Homeostasis. Cell Rep..

[137] Meli V.S., Atcha H., Veerasubramanian P.K., Nagalla R.R., Luu T.U., Chen E.Y., Guerrero-Juarez C.F., Yamaga K., Pandori W., Hsieh J.Y. (2020). YAP-Mediated Mechanotransduction Tunes the Macrophage Inflammatory Response. Sci. Adv..

[138] Zhao C., Qiu P., Li M., Liang K., Tang Z., Chen P., Zhang J., Fan S., Lin X. (2021). The Spatial Form Periosteal-Bone Complex Promotes Bone Regeneration by Coordinating Macrophage Polarization and Osteogenic-Angiogenic Events. Mater. Today Bio.

[139] Lian M., Sun B., Han Y., Yu B., Xin W., Xu R., Ni B., Jiang W., Hao Y., Zhang X. (2021). A Low-Temperature-Printed Hierarchical Porous Sponge-like Scaffold That Promotes Cell-Material Interaction and Modulates Paracrine Activity of MSCs for Vascularized Bone Regeneration. Biomaterials.

[140] Caire R., Dalix E., Chafchafi M., Thomas M., Linossier M.T., Normand M., Guignandon A., Vico L., Marotte H. (2022). YAP Transcriptional Activity Dictates Cell Response to TNF In Vitro. Front. Immunol..

[141] Liu M., Yan M., Lv H., Wang B., Lv X., Zhang H., Xiang S., Du J., Liu T., Tian Y. (2020). Macrophage K63-Linked Ubiquitination of YAP Promotes Its Nuclear Localization and Exacerbates Atherosclerosis. Cell Rep..

[142] Crockett J.C., Schütze N., Tosh D., Jatzke S., Duthie A., Jakob F., Rogers M.J. (2007). The Matricellular Protein CYR61 Inhibits Osteoclastogenesis by a Mechanism Independent of Alphavbeta3 and Alphavbeta5. Endocrinology.

[143] Zhao L., Guan H., Song C., Wang Y., Liu C., Cai C., Zhu H., Liu H., Zhao L., Xiao J. (2018). YAP1 Is Essential for Osteoclastogenesis through a TEADs-Dependent Mechanism. Bone.

[144] Xiong J., Almeida M., O’Brien C.A. (2018). The YAP/TAZ Transcriptional Co-Activators Have Opposing Effects at Different Stages of Osteoblast Differentiation. Bone.

[145] Wang L., You X., Lotinun S., Zhang L., Wu N., Zou W. (2020). Mechanical Sensing Protein PIEZO1 Regulates Bone Homeostasis via Osteoblast-Osteoclast Crosstalk. Nat. Commun..

